# Supercritical CO_2_ Extraction and Characterization of Lipophilic Natural Products from *Cornus mas* Fruits: Composition and Antimicrobial Activity

**DOI:** 10.3390/molecules31162757

**Published:** 2026-08-08

**Authors:** Elisa Visentin, Nicola De Zordi, Angelo Cortesi, Ramona Iseppi, Eva Pericolini, Cristina Forzato

**Affiliations:** 1 Dipartimento di Scienze Chimiche e Farmaceutiche, Università degli Studi di Trieste, Via L. Giorgieri 1, 34127 Trieste, Italy; elisa.visentin2@phd.units.it; 2 Società Agricola Moldoi s.r.l., Località Maras Moldoi, n.151/A, 32037 Sospirolo, Italy; 3 Dipartimento di Ingegneria e Architettura, Università degli Studi di Trieste, Via A. Valerio 10, 34127 Trieste, Italy; angelo.cortesi@dia.units.it; 4 Dipartimento di Scienze della Vita, Università degli Studi di Modena e Reggio Emilia, Via Campi 287, 41125 Modena, Italy; ramona.iseppi@unimore.it; 5 Dipartimento Chirurgico, Medico, Odontoiatrico e di Scienze Morfologiche con Interesse Trapiantologico, Oncologico e di Medicina Rigenerativa, Via Campi 287, 41125 Modena, Italy; eva.pericolini@unimore.it

**Keywords:** supercritical fluids, extraction, *Cornus mas*, HRGC, fatty acids, antimicrobial activity

## Abstract

*Cornus mas* L. fruits are a valuable source of bioactive natural products with potential applications in functional foods and food preservation. In the present study, supercritical carbon dioxide (Sc-CO_2_) extraction was applied for the recovery of lipophilic fractions from *Cornus mas* fruits under different operating conditions of pressure, temperature, and extraction time. The influence of extraction parameters on the extraction yield was investigated using a design of experiments (DoE) approach. The chemical composition of the obtained extracts was characterized via high-resolution gas chromatography (HRGC), revealing a fatty acid profile dominated by linoleic acid (58–63%) and oleic acid (22–24%), followed by palmitic acid (8–9%), stearic acid (2–4%), arachidic acid (about 2%), and behenic acid (about 1%). The extracts were further evaluated for antimicrobial activity against selected Gram-positive and Gram-negative bacteria, including *Staphylococcus aureus*, *Escherichia coli*, and *Pseudomonas aeruginosa*, as well as against the yeast *Candida albicans*. The results demonstrated that supercritical CO_2_ extraction represents an effective green technology for the selective recovery of lipophilic bioactive compounds from *Cornus mas* fruits. The extracts exhibited measurable antimicrobial activity in broth microdilution assays against selected Gram-positive and Gram-negative bacteria, as well as *Candida albicans*, supporting their potential as natural antimicrobial agents. The combination of green extraction technology, analytical characterization, and bioactivity evaluation contributes to the valorization of *Cornus mas* fruits as a source of food-derived natural products.

## 1. Introduction

**Figure 1:**
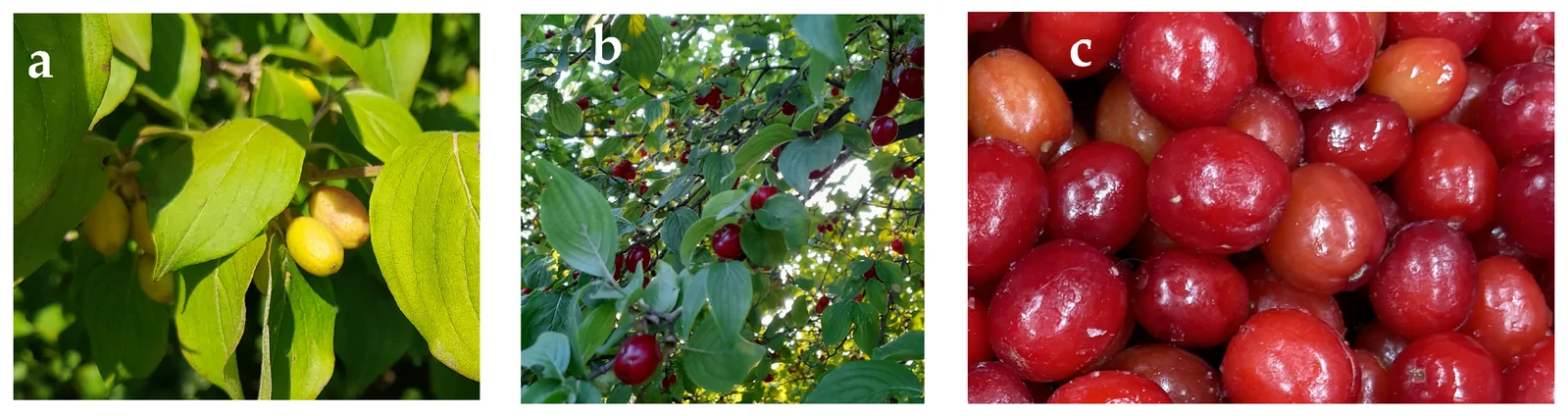
(**a**) The plant in Basovizza with fruits at the early stage of ripeness; (**b**) the plant in Basovizza with fruits at the right stage of ripeness; (**c**) morphological features of ripe *Cornus mas* L. fruits.

*Cornus mas* L. (CM, *Cornaceae*), commonly known as cornelian cherry, is a deciduous shrub widely distributed in southern and central Europe, as well as in southwest Asia, particularly in Iran. According to recent studies, 28 horticultural forms and variants of *Cornus mas* L. have been identified [1]. The edible fruits are olive-shaped and turn dark red during ripening. Fully ripened fruits (which represent the specific maturation stage investigated in this work) are represented in Figure 1c. They can be consumed fresh or processed into juices, syrups, jams, and dried products. CM fruits have attracted increasing scientific interest because of their antioxidant and antimicrobial properties, which are mainly associated with their contents of bioactive phytochemicals. The fruits contain several secondary metabolites, including anthocyanins, phenolic compounds, flavonoids, ascorbic acid, tartaric acid, malic acid, citric acid, gallic acid, loganin, and chlorogenic acid, although the concentration of these compounds may vary depending on the genotype, geographical origin, climatic conditions, and fruit ripening stage [2]. In addition, CM fruits are particularly rich in vitamin C, with levels reported to be higher than those found in oranges, strawberries, and kiwifruit [3]. These compounds are considered valuable natural products for the development of functional food ingredients and natural preservatives [4,5,6]. Several biological properties have been shown for CM fruits, as reported by Byram and Ozturkcan in 2020 in a review presenting the bioactive components and biological and therapeutic effects of fruits, leaves, seeds, and barks of CM, while providing an overview of its practicality in the pharmaceutical industry and as a nutritional supplement in the food industry [7]. The pharmacological potential of CM and other *Cornus* species has been also analyzed by Tenuta et al. in a recent review [8].

On a global scale, CM is recognized as a high-potential, underutilized fruit crop whose commercial cultivation and structured harvesting are heavily centered in Southwestern Asia and Eastern Europe [1]. There is no official data on the global scale of production of the cornelian cherry. The Turkish Statistical Institute (TÜİK) is one of the few government agencies in the world that officially monitors cornelian cherry (locally known as “kızılcık”) production. Turkish agricultural censuses record national production (which generally fluctuates between 10,000 and 14,000 tons per year), tracking both the number of cultivated trees and the output of individual provinces [7]. Conversely, in the Italian peninsula, CM (locally known as “corniolo”) currently lacks a formal commercial framework and is primarily relegated to spontaneous woodland growth or micro-scale artisanal foraging [9]. Currently, only a minimal number of small-scale organic bio-farms engage with the species for niche regional markets. However, driven by modern functional food trends and the global “rediscovery” of bioactive minor fruits, CM represents an exceptionally valuable candidate for agricultural recovery in Italy. Due to its remarkable pedoclimatic rusticity, low nutrient input requirements, high resilience to thermal fluctuations, and natural resistance to major pests, the recovery and systemic cultivation of this species offers a strategic opportunity for expanding organic agricultural systems, preserving agrobiodiversity, and economically revitalizing marginal or abandoned hilly and mountainous Italian farmlands [1,10].

Different extraction techniques and solvents can be employed for the recovery of secondary metabolites from plant matrices, and the selectivity of the process strongly depends on the extraction conditions and solvent properties. Conventional Soxhlet extraction is commonly used for the recovery of lipophilic fractions using organic solvents such as *n*-hexane, toluene, chloroform, or acetone. However, the toxicity of these solvents, together with their environmental impact and solvent residues, limits their application in food-related products. In recent years, green extraction technologies based on supercritical carbon dioxide (Sc-CO_2_) have gained increasing attention for the recovery of natural products from food matrices because they avoid toxic organic solvents and operate under mild thermal conditions [11]. Supercritical fluid extraction is particularly suitable for food applications because it enables the selective extraction of lipophilic bioactive compounds while preserving the thermolabile constituents. Furthermore, the tunable solvating power of supercritical CO_2_ allows the modulation of the extraction selectivity through changes in pressure and temperature. Despite the growing interest in CM fruits, the application of Sc-CO_2_ extraction to this plant material is still poorly investigated. Jakovljević et al. studied the supercritical CO_2_ extraction of cornelian cherry seeds using different extraction parameters such as pressure and temperature, and the optimal extraction conditions were determined via response surface methodology (RSM) using a central composite rotatable design (CCRD) [12]. The composition of the extracted seed oil was also reported. More recently, Gillani et al. reported the Sc-CO_2_ extraction of CM [13]; however, only the extraction yield under a single operating condition was investigated. In the present study, the influence of the extraction pressure (P), temperature (T), and extraction time (t) on the supercritical CO_2_ extraction of CM fruits was investigated using a design of experiments (DoE) approach. Compared with previous studies, the present work introduces a more comprehensive experimental design by including the extraction time as an additional variable, allowing a more accurate description of the extraction system. In addition, the chemical composition of each extract was characterized via high-resolution gas chromatography (HRGC) in order to evaluate the influence of extraction conditions on the qualitative and quantitative profile of the recovered lipophilic fraction. Furthermore, the antimicrobial activity of the extracts was investigated against selected microorganisms in order to evaluate their potential as natural antimicrobial agents for food-related applications. The combination of green extraction technology, analytical characterization, and bioactivity evaluation provides useful information for the valorization of CM fruits as a source of food-derived natural products.

## 2. Results and Discussion

### 2.1. Sc-CO_2_ Extraction Optimization

RSM is a method that, through the regression analysis of experimental data, allows a relationship between an analyzed response value (yield %) and the various factors that influence it, such as pressure, temperature, and time, to be defined through the generation of a mathematical model [14,15].

The operating ranges examined for the three variables were selected considering three main criteria: the technical limitations of the extraction unit, the need to maintain conditions within a range suitable for keeping the CO_2_ in the supercritical state while preserving any thermolabile compounds present in the extracted oil, and the operating conditions previously reported in the literature for CM and other drupe matrices (Table 1). Given the limited literature available specifically on the extraction of CM fruits, the operating parameters, extraction yields, and general processing conditions were compared with those of other similar fruit stones and seeds (*Prunus* species, Table 1).

The pressure was studied in a range between 110 and 310 bar in order to cover a wide range of Sc-CO_2_ densities while remaining compatible with the safe operating limits of the extraction system. The temperature range (32–52 °C) was chosen to ensure supercritical CO_2_ conditions, avoiding excessively high temperatures (which in some reported studies reach up to 60–70 °C) that could negatively affect any thermolabile constituents present in the oil. The selected extraction time interval (1 and 6 h) allowed both the initial and later stages of the extraction process to be investigated within a practically achievable operating range. No preliminary kinetic experiments were performed to define this range, since the objective of this work is to evaluate the combined influence of pressure, temperature, and extraction time on the extraction yield.

The results of the DoE experiments are shown in Table 2. The five central point replicate experiments (runs 15–19) allow the estimation of process reproducibility and experimental variability, producing an average extraction yield of 2.17 ± 0.20% (mean ± SD), corresponding to a relative standard deviation (RSD) of 9.17%.

The extraction yield ranges from 0.40% (Line 13) to 2.93% (Line 8). Furthermore, 10 out of the 15 evaluated conditions yielded higher extraction values than those obtained with the Soxhlet extraction using *n*-hexane (1.55%). The data shown in Table 2 were analyzed, using variables such as the coded level, based on the fit following the RSM of the experimental yields with Equation (9) (see experimental Section 3.4), to investigate the factors that influence the yield and understand the trend in its variation.

Polynomial regression analysis then made it possible to obtain the constant and the linear coefficients for each variable, for the quadratic term, and for the interaction between variables. The regression coefficients obtained from the RSM analysis are shown in Table 3 with their respective p-values, where the coefficients significant at a 95% confidence level are shown in bold.

The result obtained by fitting Equation (9) is summarized in Equation (1) (R^2^ = 0.8890).

(1) $$\begin{array}{cc} Y=2.1616\;+ & 0.3504x_{1}+0.1771x_{2}+0.6345x_{3}-0.0614x_{1}^{2}-0.1144x_{2}^{2}-0.1816x_{3}^{2}+0.2300x_{1}x_{2}-0.1550x_{1}x_{3}\\ & \;-0.1500x_{2}x_{3} \end{array}$$

Each variable in Equation (1) is expressed as defined in Table 4 by the coded values.

**Figure 2:**
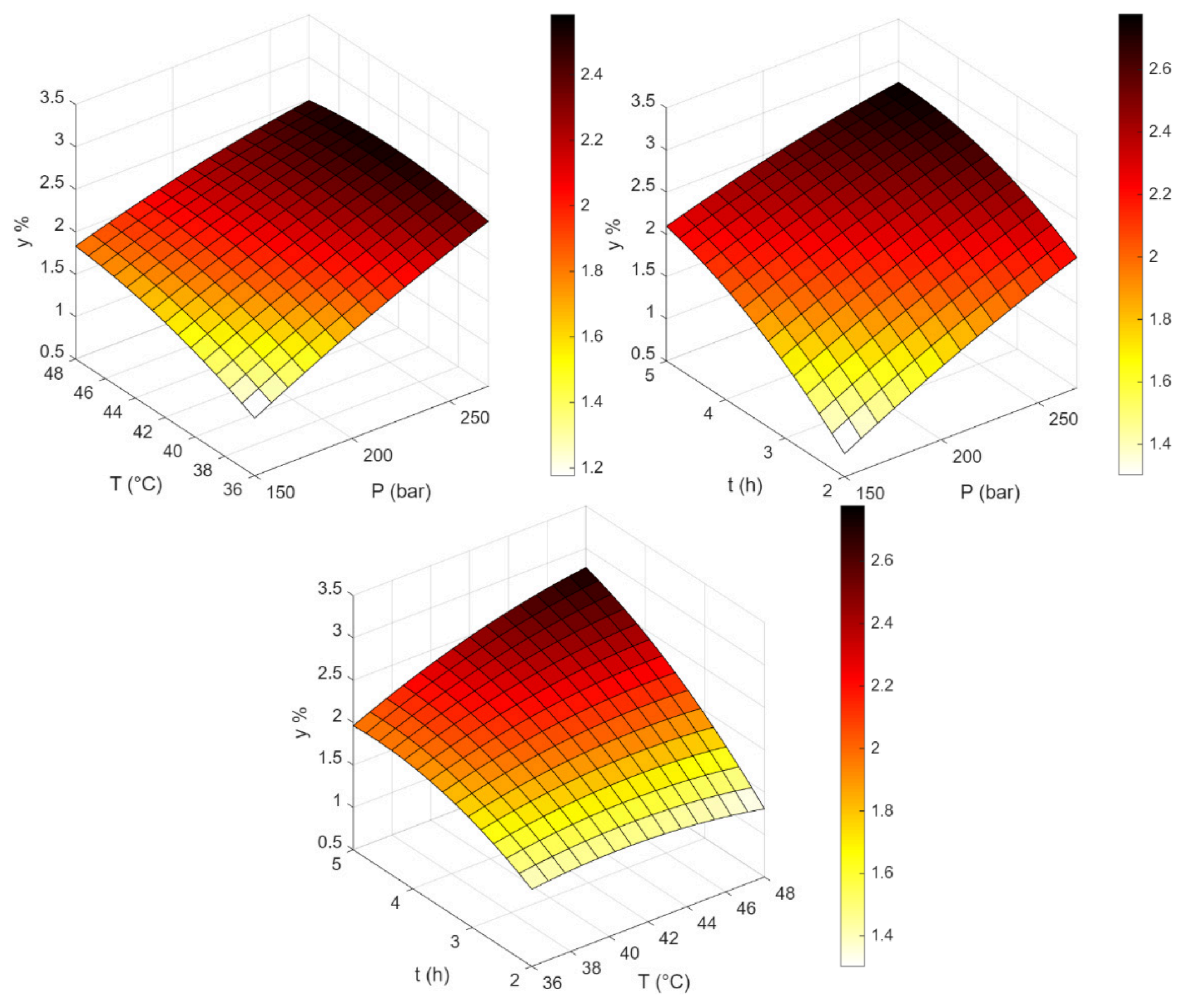
Generated surface: y% surface area.

Since a low *p*-value (≤0.05) indicates a significant term, according to Table 3, it can be inferred that a statistically significant effect on the linear increase in yield is given by an increase in pressure and, to a greater extent, by an increase in the extraction time, while no linear effect on the response was observed due to temperature variation. Quadratic effects or interactions between variables are not significant at a 95% confidence level. The greater influence of the pressure and time variables on the extraction yield can also be seen in Figure 2, which shows the response surfaces for the extraction yield, each as a function of two parameters (e.g., pressure and temperature), where the third parameter is kept constant at the point in the coded value 0 (e.g., time, 3.5 h). The results obtained indicate that the extraction yield increases with an increase in pressure and time, while the effect is weaker in the case of an increase in temperature, especially at low pressures, with a more marked effect at higher pressures.

These observations are justified by the fact that, as the pressure increases under isothermal conditions, there is a corresponding increase in the density of supercritical CO_2_, resulting in an increase in solvent power and solubility of the compounds. On the other hand, an increase in temperature at a given pressure leads to a decrease in density with a corresponding decrease in solvent power. Therefore, many factors can simultaneously influence the extraction process, making the influences of each variable complex.

The graphs in Figure 2 show a continuous increase in yield as the parameters considered increase, with no evidence of reaching a plateau. This behavior suggests that, within the range explored, the system has not yet reached an asymptotic extraction regime. (i.e., extraction equilibrium was not reached within 5 h, and the yield was still increasing at the end of the process). The best predicted conditions within the investigated range for the pressure, temperature, and extraction time (coded levels +1, +1, and +0.90, respectively) are in fact close to the vertex of the cube corresponding to the maximum values of the experimental domain (+1, +1, +1). The quadratic model (Equation (1)) predicted a maximum yield of 2.89% under conditions of 270 bar, 48 °C, and 4.85 h. In order to experimentally validate the model prediction, two tests were conducted under these conditions, obtaining a yield equal to 2.96% and 2.89%, respectively, for extract 20 and 21, reported in Table 2.

**Figure 3:**
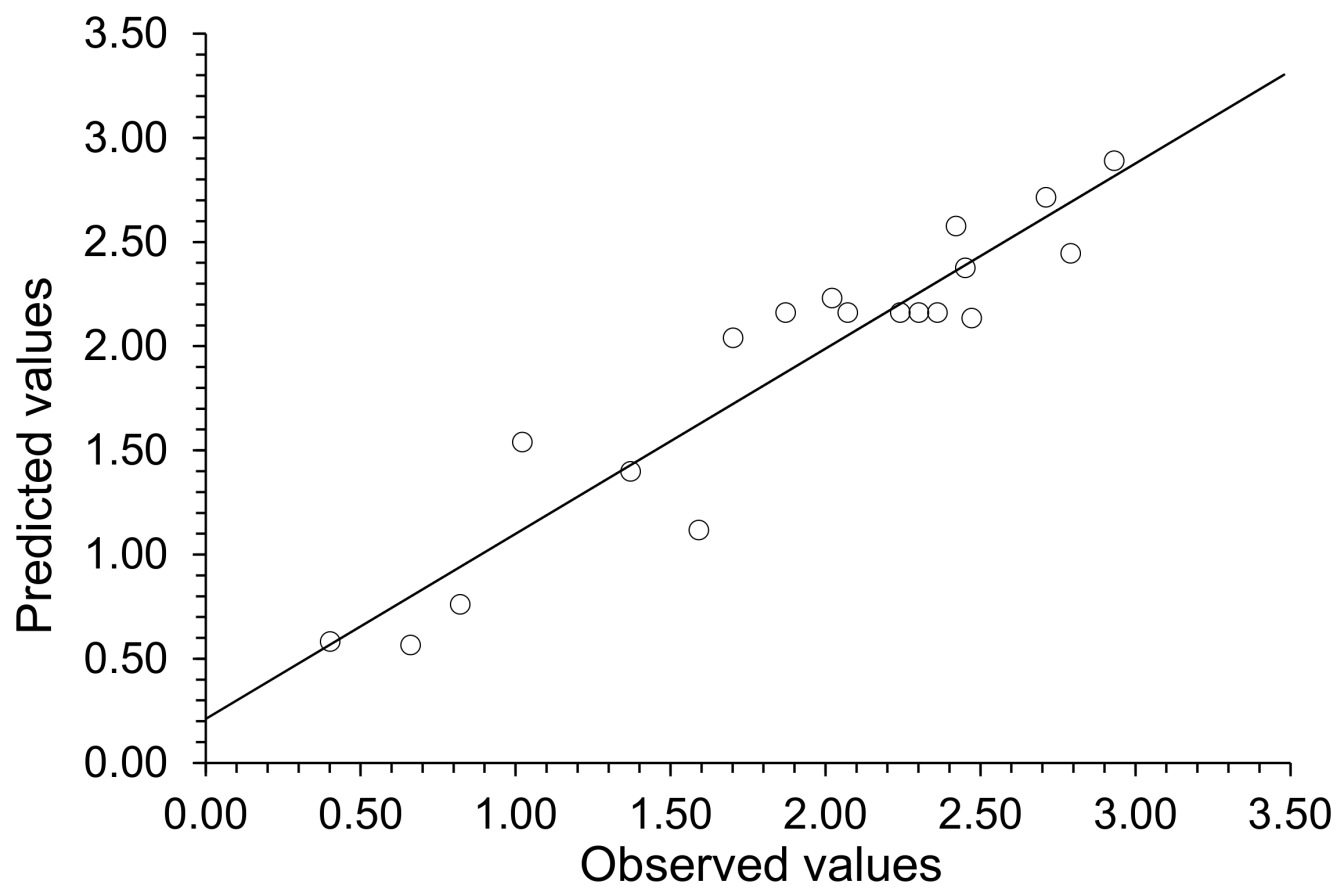
GOF graph, predicted vs. observed values (y%).

With the model obtained, 88.9% of the variability in the extraction yield is explained by Equation (1). This capability of the model is also confirmed by the graph reported in Figure 3, where the goodness of fit (GOF) is reported. In the GOF graph, the predicted values are reported as a function of the observed values for the extraction yield. What can be observed is that the points are close to the diagonal (R^2^ = 0.8888), indicating the model’s ability to fit the experimental data.

Despite this, the calculation of R^2^_adj_ (0.7780) gives a lower correlation value, which, together with a negative R^2^_pred_ (−0.1968), confirms the overfitting related to the complexity of the model. This further proves that quadratic and interaction terms are not making a significant contribution. Therefore, a reduced model (Equation (2)) that includes only the constant and statistically significant terms (x_1_ and x_3_) was selected as final predictive model to improve the robustness and generalization capacity:

(2) $$Y=\alpha_{0}+{\alpha_{1}x}_{1}+\alpha_{3}x_{3}$$

A regression analysis designed in this way allows a new constant and linear coefficients for the variables pressure and extraction time to be obtained, as reported in Table 5.

The result obtained from fitting Equation (2) can be summarized with the following equation (R^2^ = 0.7166):

(3) $$Y=1.9047+0.3504x_{1}+0.6345x_{3}$$

After removing the non-significant terms, the reduced model shows a reduction in R^2^ (0.7166), compared to what was observed for the quadratic model; however, this is expected behavior, as the coefficient of determination is naturally influenced by the number of variables in the model [15]. The coefficient of determination alone cannot be used as a descriptor of the model’s goodness of fit. Rather, what should be noted is that, for the reduced model, there is a closer agreement among R^2^, R^2^_adj_ (0.6755), and R^2^_pred_ (0.6223), confirming its high reliability.

In particular, the transition to a positive R^2^_pred_ (0.6223) demonstrates that the removal of non-significant terms has ensured a better ability to generalize and predict results obtainable from new data (Table 6), making the reduced model statistically more reliable.

The reduced model (Equation (3)), which includes only first-order terms for pressure (x_1_) and time (x_3_), shows a linear relationship between the considered variables and the extraction yield. According to this model, the yield increases strictly without reaching a plateau within the investigated range; consequently, according to Equation (3), the best predicted extraction conditions shift exactly to the extreme upper vertex of the experimental domain (270 bar and 5 h, coded levels +1 and +1), predicting a maximum yield of 2.89%. Both models converge on nearly identical yield predictions at consistent optimized coordinates, confirming that model reduction preserved the true physical location of the optimum while successfully eliminating uninformative statistical variance and restoring predictive reliability. Although the temperature of 48 °C (identified as the best condition within the investigated range by the model described in Equation (1)) successfully validated the model through the results obtained for extracts 20 and 21, the reduced model (Equation (3)) demonstrates that temperature is not a significant factor in determining the yield within the evaluated experimental domain. This implies that the possible choice to operate at lower temperatures within the evaluated domain represents a viable, energy-efficient option which, as predicted by the model, does not significantly affect the extraction yield.

### 2.2. Chemical Profile of the Extracts

#### 2.2.1. NMR Analysis

**Figure 4:**
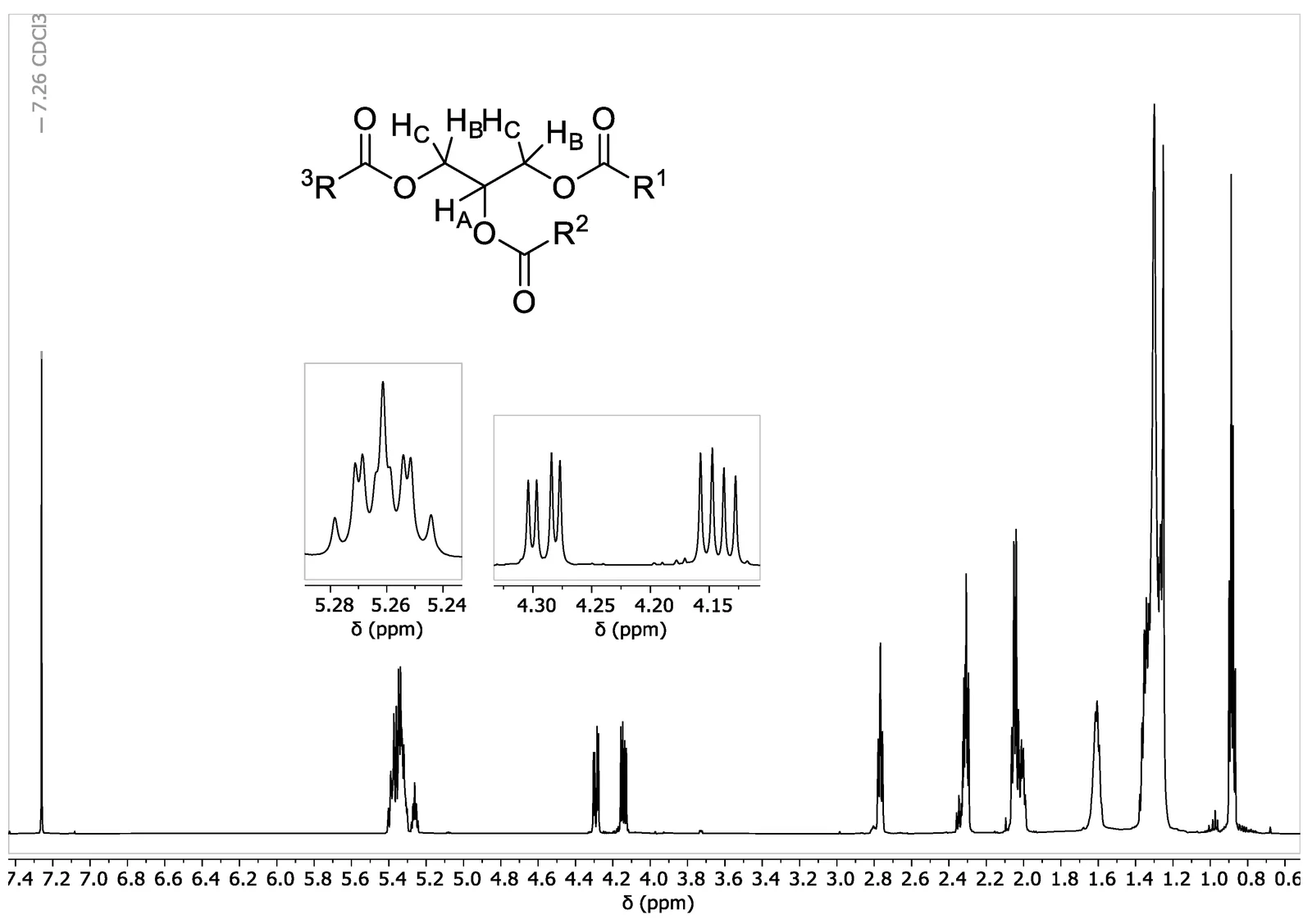
^1^HNMR spectra of extract No. 20 in CDCl_3_ and chemical structure of a triacylglyceride; R^1^, R^2^, and R^3^ are alkyl chains.

**Figure 5:**
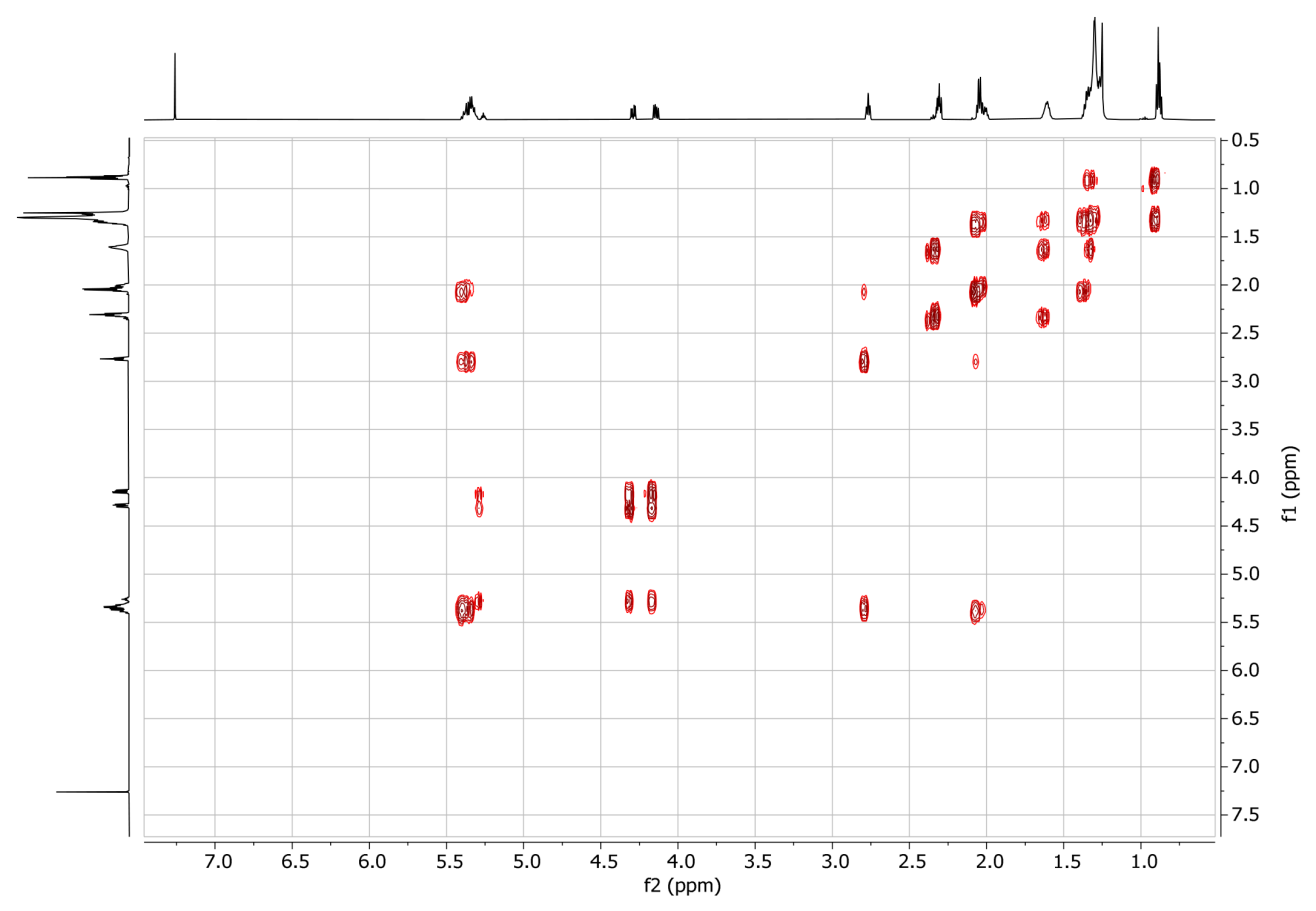
^1^H-^1^H COSY spectra of extract No. 20 in CDCl_3_.

**Figure 6:**
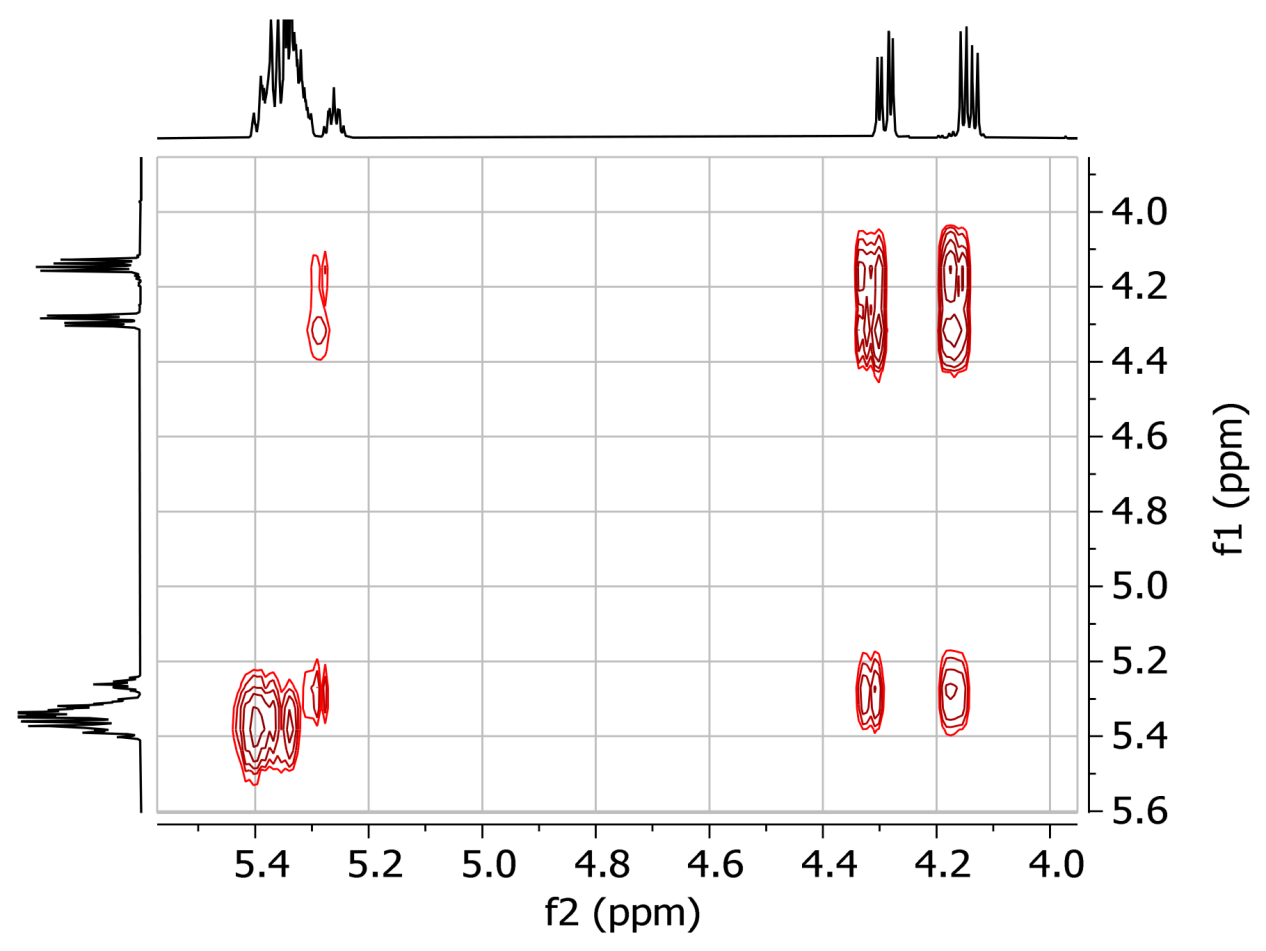
Zoomed-in region of the ^1^H-^1^H COSY spectra of extract No. 20 in CDCl_3_.

**Figure 7:**
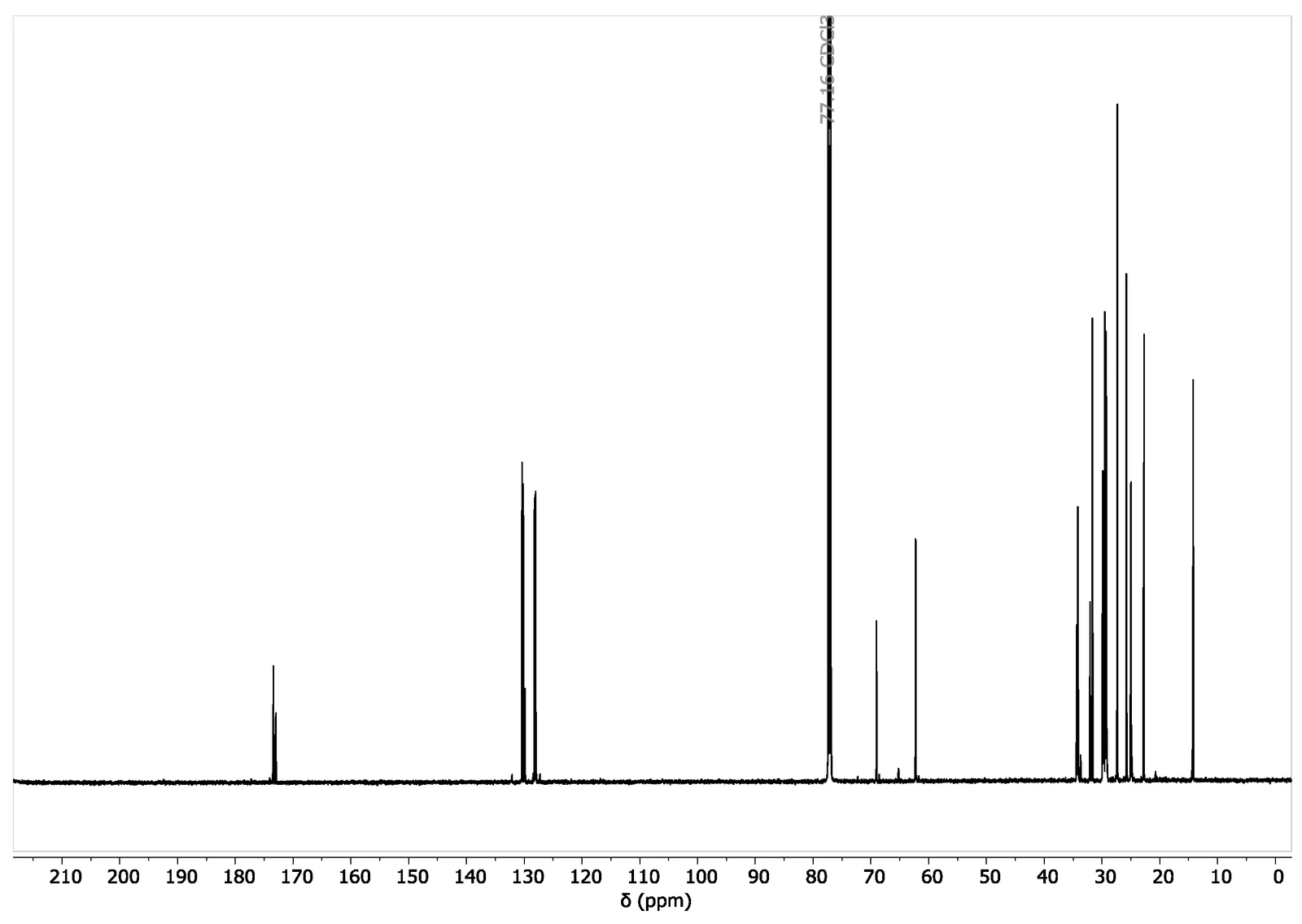
^13^CNMR spectra of extract No. 20 in CDCl_3_.

The ^1^HNMR analysis was included to provide a non-destructive fingerprint of the intact CM extracts, complementing the HRGC data reported in Section 2.2.2. The ^1^HNMR and ^13^CNMR spectra of the crude extracts revealed that triacylglycerides, comprising both saturated and unsaturated fatty acids, represent the predominant class of compounds. While HRGC quantifies individual fatty acids after transesterification, ^1^HNMR validates the native lipid architecture, confirming that the fatty acids are bound as triacylglycerols. As an example, in Figure 4, the ^1^HNMR spectra of extract No. 20 is reported, where the typical signal pattern for glycerol can be recognized, with the methylene protons H_B_ and H_C_ at 4.29 and 4.14 ppm as double doublets having the geminal and vicinal couplings ^2^J = 11.9 Hz, ^3^J = 6.0 and 4.3 Hz, respectively, and the signal of the methine proton H_A_ at 5.26 ppm as a triplet of triplets, having the vicinal coupling constants ^3^J = 5.9 and 4.3 Hz. This coupling is also observed in the ^1^H-^1^H COSY spectrum (Figure 5 and Figure 6), where cross-peaks are visible due to the coupling between the geminal methylene protons with each other and with the methine proton. ^13^CNMR shows signals characteristic of the carbonyl carbons of esters (172.99, 173.42, and 173.43 ppm) (Figure 7). Different fatty acids are present in the triacylglyceride structure since several protons are present in the range between 0.8 and 2.8 ppm. Moreover, signals between 5.30 and 5.40 ppm are also observed, corresponding to vinylic protons and indicating the presence of unsaturated fatty acids.

From the analysis of ^1^HNMR, it was also possible to identify signals in the spectrum corresponding to vinyl protons and allyl protons, which resonate at different fields depending on whether they belong to mono-, di-, or tri-unsaturated fatty acids, as reported by Salinero et al. [20]. The presence of bisallylic protons belonging to di-unsaturated (2.78–2.74 ppm) and tri-unsaturated (2.81–2.78 ppm) fatty acids suggests the possible presence of linoleic acid (C18:2) and linolenic acid (C18:3), respectively. Based on the results reported in the aforementioned study, Table 7 shows the assignments for the signals at different chemical shifts and their areas (assigning area 1.000 to the methine proton) for extract No. 20.

In particular, the integral areas of signals 6, 7, and 11 in the ^1^HNMR spectrum can be used to obtain the distribution of saturated, mono-unsaturated, di-unsaturated, and tri-unsaturated fatty acids present in the triacylglycerides. Salinero et al. proposed a series of equations to determine the percentage of the different classes of fatty acids present in the triacyglycerides (Equations (4)–(7)) [20]. By substituting the integral signal areas reported in Table 7 into the system of Equations (4)–(7) and reporting the results as a percentage, it is possible to obtain, for extract No. 20, the molar percentages reported in Table 8.

(4) Vynilic H integral=2x+4y+6z

(5) $$Bisallylic\;H\;integral\left(di-unsaturated\;fatty\;acid\right)=2y$$

(6) $$Bisallylis\;H\;integral\left(tri-unsaturated\;fatty\;acid\right)=4z$$

(7) x+y+z+w=3

X, y, z, and w represent the moles of mono-, di-, and tri-unsaturated and saturated fatty acids per mole of triglyceride, respectively. The area of the integral of vinyl protons corresponds to the area of peak 11, while those of bisallylic protons correspond to the area of peaks 6 and 7 for di-unsaturated and tri-unsaturated fatty acids, respectively.

Although ^1^HNMR spectroscopy provided a rapid and non-destructive tool for compound class identification and the estimation of fatty acid saturation classes, HRGC analysis (as described in the following section) was subsequently performed to obtain the molecular speciation and precise quantification of individual fatty acids. However, NMR alone cannot exclude the presence of other minor lipophilic constituents in the extract below the detection limit. For these minor components, complementary techniques, such as LC-MS or GC-MS, could provide additional information in future investigations.

#### 2.2.2. HRGC Analysis

To determine the composition of fatty acids by means of HRCG, a transesterification reaction of the extracts with methanol has been performed since fatty acids are present as triglycerides. The fatty acids obtained are present as methyl esters fatty acids (FAMEs). The identification via HRGC was possible using, as a standard, a mixture of 14 fatty acid methyl esters. The relative quantification of individual fatty acids in the CM oils was successfully performed through HRGC-FID analysis of the corresponding methyl esters (FAMEs), identifying each compound based on a comparison with a standard reference mixture and calculating mass percentages based on specific response factors Rf_i_. A full validation of the analytical method (such as absolute recovery and inter-day reproducibility studies) was not performed, as the primary objective of this work was the relative compositional profiling (%*w*/*w*) of fatty acids, rather than the validation of a new quantitative method.

**Figure 8:**
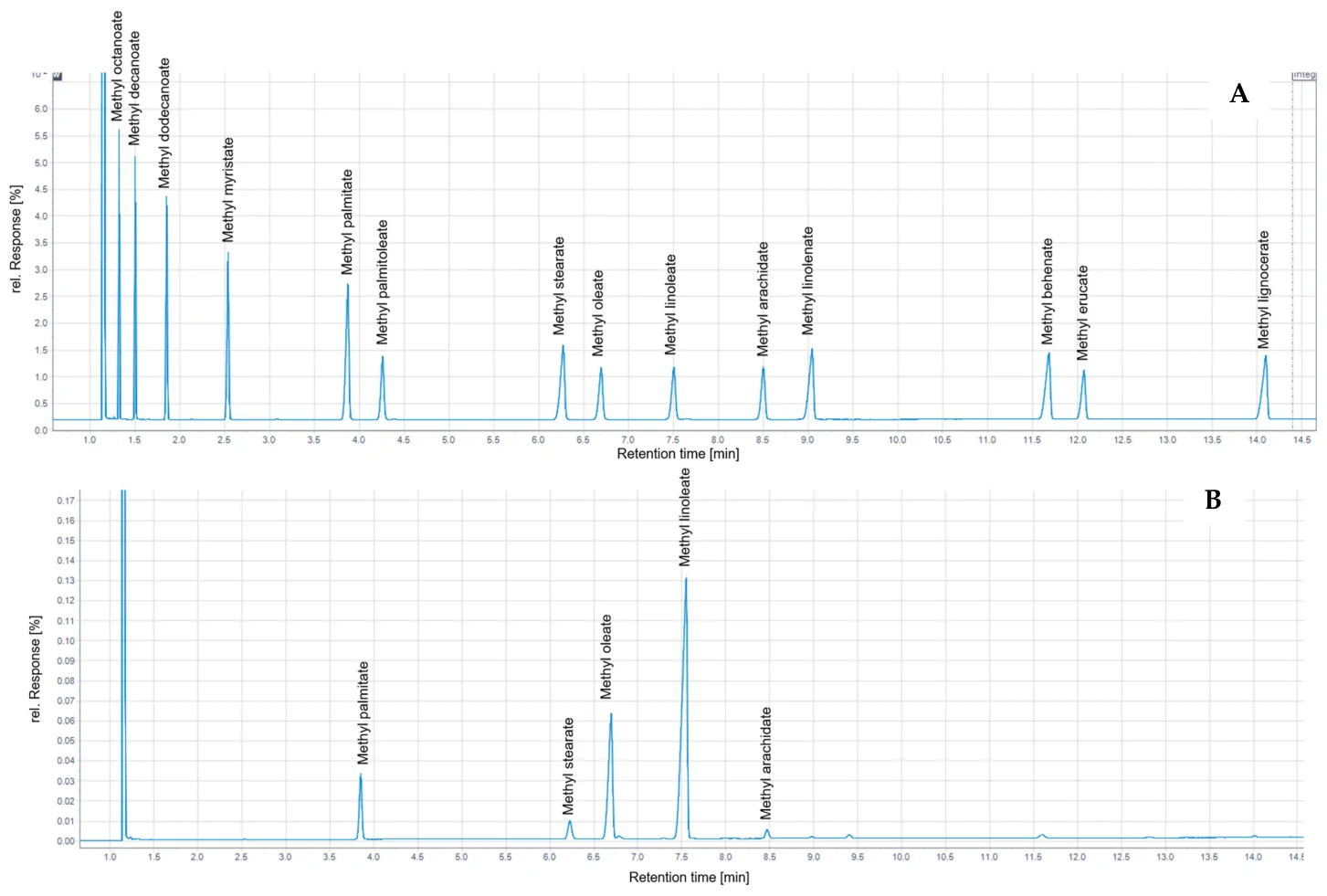
Chromatogram of the FAME mixture standard (**A**) and chromatogram of extract No. 15 (**B**).

The composition of fatty acids identified via HRCG in each extract performed via Sc-CO_2_ extraction is reported in Table 9. The FAME profile for the Soxhlet extraction and for extractions carried out at the best predicted extraction conditions within the investigated range of the DoE model are also included. In Figure 8A, the chromatogram of the standard mixture of FAME is shown, while in Figure 8B, the chromatogram of extract No. 15 is reported as an example. As shown in Table 9, the fatty acid composition of the oils obtained under different extraction conditions using Sc-CO_2_ falls within a narrow range, exhibiting a profile closely comparable to that obtained via traditional Soxhlet extraction. In general, it can be observed that unsaturated fatty acids are more abundant with respect to the saturated fatty acids in all extracts. Linoleic acid is always the most abundant fatty acid (C18:2) with a percentage ranging from 59.759% to 63.168%. A significant amount of oleic acid (C18:1) was also determined with a percentage in the range 22.189–23.550%, while palmitic acid (C16:0) was quantified in the range 7.569–9.066%. These results are in accordance with those reported for the supercritical extract of CM seeds by Jakovljević et al. in 2018 [12].

Significant amounts of stearic acid (2.270–4.185%), arachidic acid (2.218–2.436%), and behenic acid (0.595–1.362%) were also determined, while myristic acid, linolenic acid, and lignoceric acid were always present in all extracts, but in very low amounts (<1%). All the other fatty acids were present only in traces. This composition reflects the lipid profile of *Cornus mas* rather than the effect of the selective supercritical CO_2_ extraction. This is confirmed by the comparison with Soxhlet extraction, using *n*-hexane as a non-polar solvent with solubility parameters comparable to Sc-CO_2_. The main components of the extracted oil are also, in this case, linoleic acid (58.451%), oleic acid (23.618%), and palmitic acid (9.509%), and the percentages determined are similar to the ones determined for Sc-CO_2_ extraction. Furthermore, the values reported in Table 9 fall within the range reported in the literature for CM seed oils (linoleic acid: 60.2–75.0%, oleic acid: 15.7–23.7%, palmitic acid: 3.5–8.1%) [21]. Extractions performed at the optimum point gave values in the range observed for the other extractions, but the yield of extraction was significantly higher.

While variations in the pressure and extraction time significantly affected the overall extraction yield, the fatty acid composition varied within a narrow range across all experiments. This behavior can be attributed to the nature of the extracted compounds and thermodynamic properties of the solvent. As discussed above, CM extracts are predominantly composed of triacylglycerols, consisting primarily of C16 and C18 fatty acid chains. Therefore, since the main triacylglycerols exhibit very subtle differences in molecular weight, polarity, and vapor pressure over the pressure and temperature range studied, over the pressure and temperature range studied, variations in the Sc-CO_2_ density primarily affected its overall solvating capacity, increasing the extraction yield and preserving the natural fatty acid profile of the seed without inducing selectivity.

The relative abundances of fatty acids grouped by saturation class from the HRGC analysis were compared with preliminary NMR predictions. Since HRGC provides mass percentages while NMR provides molar percentages, the HRGC mass data for extract 20 were mathematically converted to molar percentages and grouped by fatty acid saturation class, to allow a direct comparison of results obtained using the two techniques. The two techniques showed agreement in the observed results; for example, for extract 20, the converted HRGC data determined a composition of 14.50% saturated fatty acids, 22.83% mono-unsaturated fatty acids, and 62.50% di-unsaturated fatty acids, closely matching the percentages predicted by NMR (13.72%, 21.47%, and 62.72%, respectively, reported in Table 8).

This comparison confirms that NMR spectroscopy is a reliable and non-destructive method for predicting the composition of total fatty acids in triglycerides, while HRGC provides a more detailed profile, allowing the qualitative determination of fatty acids and providing quantitative information.

### 2.3. Antimicrobial Activity of the Extracts

#### 2.3.1. Disk Diffusion Assay

The agar disk diffusion assay was used for an initial qualitative assessment of the activity of two extracts of *Cornus mas* L. against the indicator microbial strains. Extracts 23 and 24 were specifically selected for antimicrobial screening as representative composite samples. Extract 23 was prepared by pooling the replicates of the central point (runs 15–19, Table 9), whereas extract 24 combined the runs performed using the predicted optimum conditions (runs 20–21, Table 9). These pooled extracts provided a sufficient and highly representative volume of the typical CM lipid profile required to perform the complete panel of microbiological assays. Amphotericin B and Levofloxacin were used as positive controls. A growth inhibition zone was observed only for *E. coli* using extract 23 (8 mm ± 0.4). Both the antifungal (Amphotericin B) and antibiotic (Levofloxacin) exhibited strong activity, with an inhibition zone of 15 mm ± 1.3 for *C. albicans* and a range of 25 to 30 mm (±0.6–1.1) for bacteria (Table 10). The antimicrobial activity of lipophilic extracts evaluated through agar diffusion assays should be interpreted with caution, since the limited diffusion of hydrophobic compounds through the agar matrix may underestimate their inhibitory potential. For this reason, the disk diffusion assay was employed only as a preliminary qualitative screening method, whereas the quantitative assessment of antimicrobial activity was based on the broth microdilution assay as reported below, which is considered more appropriate for poorly water-soluble natural extracts. Indeed, several authors have highlighted that agar diffusion methods may underestimate the activity of hydrophobic plant extracts because antimicrobial compounds diffuse poorly through the agar medium [22,23].

#### 2.3.2. The Minimum Inhibitory Concentration (MIC) and Minimum Fungicidal/Bactericidal Concentration (MFC/MBC)

Both extracts 23 and 24 showed comparable antimicrobial activity against the indicator microbial strains. For all the bacterial strains, the MICs were observed at the extract dilution 1:2, while the MBC was reached at the extract dilution 1:1. The best activity of the *Cornus mas* L. oil extracts was obtained towards *C. albicans* with an MIC at the extract dilution 1:3 and MFC at the extract dilution 1:2 (Table 11). The antimicrobial assays were performed using serial dilutions of the crude extract starting from the neat extract. Therefore, MIC and MBC/MFC values are expressed as dilution ratios.

#### 2.3.3. Growth Kinetics Study

**Figure 9:**
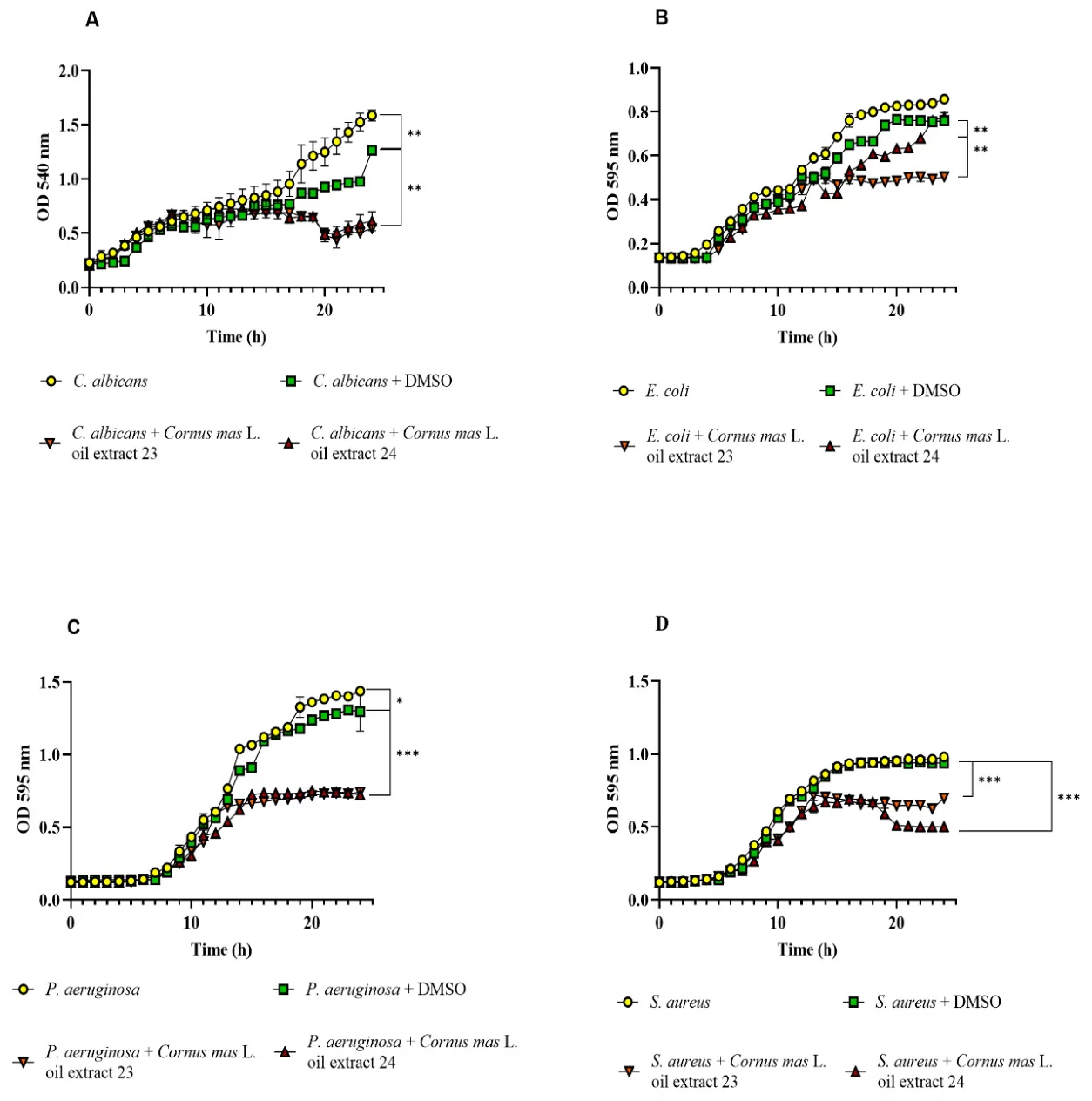
Growth kinetics study of *Cornus mas* L. extract 23 and *Cornus mas* L. extract 24 against (**A**) *C. albicans*, (**B**) *E. coli*, (**C**) *P. aeruginosa*, and (**D**) *S. aureus*. Results are expressed as the mean ± SD of the two determinations (error bar = S.D.; *n* = 2). Statistical significance differences between experimental groups were indicated as: *p* < 0.05 (*), *p* < 0.01 (**) and *p* < 0.001 (***).

For all the microorganisms, both *Cornus mas* L. extracts displayed significant anti-microbial activity, as compared to the effect of the diluent (DMSO) (Figure 9). While for *C. albicans*, *P. aeruginosa*, and *S. aureus*, the activity of the two extracts was comparable (Figure 9A,C,D), for *E. coli*, a greater antimicrobial effect was observed for extract 23 (Figure 9B). A slight but significant antimicrobial effect was also observed with the diluent against *C. albicans* and *P. aeruginosa* (Figure 9A,C). However, this effect was significantly lower than that exerted by the extracts. Although the growth kinetics experiments were performed using two independent biological replicates and should therefore be interpreted with appropriate caution, the observed trends were consistent with the results obtained using the broth microdilution assay. Consequently, the growth kinetics analysis shows complementary evidence supporting the antimicrobial activity of the extracts.

Several studies have investigated the antimicrobial properties of different parts of CM, including extracts obtained from fruits, seeds, leaves, and bark, as well as the microbiological effectiveness of seed-derived fatty oil and aqueous extracts [24,25]. CM seed oil has demonstrated strong antifungal activity, particularly against *Candida* species, and has also been shown to inhibit both Gram-positive and Gram-negative bacteria. In addition, it was shown that aqueous extracts of CM were effective against *Staphylococcus aureus*, *Bacillus subtilis*, and *Candida albicans* [25]. These antimicrobial effects are likely associated with the presence of bioactive constituents in the extracts, such as phenolic compounds and fatty acids, which are well recognized for their antimicrobial activity. In line with this evidence, we showed that CM extracts tested in this work exert antibacterial effects against Gram-positive and Gram-negative bacteria as well as an anti-fungal effect.

It is well known that fatty acids (FAs) possess antimicrobial activity towards several strains of bacteria including multidrug-resistant bacteria [26]. Moreover, they showed a synergistic effect in their antimicrobial activity in combination with different antibiotics, such as penicillins, fluoroquinolones, and aminoglycosides [27,28]. Both saturated and unsaturated FA exert this effect on Gram-negative and Gram-positive bacteria, although the results reported in the literature present some discrepancies. This is the case for palmitic acid, but the effects observed probably depend on to the use of liposomes as drug carriers, which could facilitate the delivery of palmitic acid.

Linoleic acid is the main component of CM extracts (about 60%), and this unsaturated FA has been reported, in the literature, to alter the peptidoglycan synthesis genes in *S. aureus* [29], and it also displayed potent and selective inhibitory effects on FabI in *S. aureus* and *E. coli* [30].

Oleic acid, present in CM extracts at about 23%, is reported in the literature, in its liposomal formulation, to be able to restore the susceptibility of these multidrug-resistant *P. aeruginosa* strains to the antibiotics cefepime (CPM), ciprofloxacin (CIP), ceftazidime (CAZ), ampicillin (AMP), piperacillin (PIP), cephalexin (CN), amikacin (AK), imipenem (IPM), tobramycin (TOB), gentamycin (GEN), ceftriaxone (CTX), and nitrofurantoin (NIT) [28].

Palmitic acid, present in CM at about 8%, in its liposomal formulation, is reported to display MIC values of 0.5 μg/mL towards *S. epidermis* and 2 μg/mL towards *Enterococcus faecalis* [31].

Anyway, despite the single effects that each FA can exert on different microorganisms, the synergistic effects of a mixture of FA could lead to a positive response in the antibacterial activity of a plant extract.

Compared to the aforementioned studies, which focused on the activity of free fatty acids, this study tested the activity directly on triglycerides. Therefore, the differences observed in antimicrobial activity among the extracts may be associated with differences in their overall fatty acid composition. However, since the extracts are mainly composed of triglycerides, the contribution of individual fatty acids, including linoleic acid, cannot be established from the present study. Furthermore, possible synergistic interactions among the different lipid constituents cannot be excluded. Although the extraction conditions did not substantially modify the overall fatty acid composition of the lipophilic extracts, a biological evaluation was performed on representative extracts obtained under different operating conditions in order to assess whether changes in the extraction process could influence their antimicrobial properties. The similarity of the fatty acid profiles indicates that the differences observed in antimicrobial activity cannot be directly attributed to variations in the major fatty acids alone. Instead, they may reflect the contribution of minor constituents not investigated in the present study or synergistic interactions among the different components of the lipophilic fraction. Consequently, the present study does not establish a causal relationship between individual chemical constituents and antimicrobial activity, but rather demonstrates that supercritical CO_2_ extraction conditions can yield lipophilic extracts with measurable antimicrobial potential. The activity observed for *C. albicans*, *P. aeruginosa*, and *S. aureus* is therefore not unexpected, as these microorganisms possess lipases capable of hydrolyzing triglycerides [32,33,34]. Although hydrolytic activity is less frequently reported in the literature for *E. coli*, some studies report that supercritical extracts [35] and glycerol monoglycerides [36] have inhibitory activity against this microorganism, suggesting the presence of lipid components in the extract that contribute to the observation of an antimicrobial effect.

Concerning the growth kinetics test performed on *E. coli*, different activity was observed for extracts 23 and 24, which could be attributed to differences in the compositions of the extracts. For this microorganism, the literature reports that linoleic acid and its derivatives exhibit significant antibacterial activity against *E. coli* by inhibiting growth and suppressing virulence gene expression [37]. Consistent with this, the extract showing greater activity, extract 23, contains a significantly higher percentage of linoleic acid (*p* < 0.001) compared to extract 24. However, the synergistic contribution of the other fatty acids composing the extract, which are present in different amounts, cannot be excluded; in fact, extracts 23 and 24 differ significantly not only in linoleic acid content, but also in other major constituents, such as palmitic acid (*p* < 0.001), stearic acid (*p* < 0.001), and arachidonic acid (*p* < 0.001).

## 3. Materials and Methods

### 3.1. Chemicals

Heptane (purity 99%), *n*-hexane (purity ≥ 95%), methanol (purity ≥ 99,9%), and potassium hydroxide (purity ≥ 85%) were purchased from Sigma-Aldrich (St. Louis, MO, USA); the C8–C24 fatty acid methyl ester standard mixture (CRM 18918) was purchased from Supelco (Bellefonte, PA, USA); CO_2_ was purchased from SIAD (Trieste-Italy).

### 3.2. Fruit Material

The ripe *Cornus mas* L. fruits were harvested in Basovizza—Trieste (Italy) (45°38′53.6″ N 13°50′44.8″ E)—between July and August 2024. The whole fruits were dried in an oven at 40 °C for 5 days until reaching a constant weight, allowing moisture removal (residual moisture content of <8%) without degrading thermolabile compounds. To increase its surface area for subsequent extractions and to homogenize the material, the dried fruits were then ground using a Retsch SM 100 mill (Retsch, Haan, Germany) to an average particle size of 2 mm. This particle size represented an optimal compromise, as it was observed that further grinding to a finer particle size induced excessive frictional heating, resulting in caramelization of the sugar and potential degradation of thermolabile compounds. The dry ground material was stored in the freezer at −20 °C.

### 3.3. Extraction Methods

#### 3.3.1. Soxhlet Extraction

Conventional extraction by Soxhlet using *n*-hexane was performed. An amount of 15 g of dried and ground fruit was placed in a cellulose thimble. The sample was extracted in a Soxhlet apparatus with 200 mL of *n*-hexane at reflux for 6 h. The solvent was then removed in a rotary evaporator, and the extraction yield was calculated according to the following equation:

(8) $$Y=\frac{weight\;extract}{dry\;weight\;of\;Cornus\;mas}\times 100$$

#### 3.3.2. Supercritical Carbon Dioxide Extraction

Sc-CO_2_ extractions were conducted in a Separex SFE 20 extractor (Separex, Champigneulles, France) under the established temperature and pressure conditions. A schematic diagram of the supercritical fluid extractor has been previously reported by De Zordi et al. [38]. The temperature was preset on the instrument, and the pressure was checked using a pressure manometer. The extraction equipment was connected to a high-pressure pump (Lewa EKM210V1) (Lewa, Leonberg, Germany), whose flow rate was adjusted according to the pressure to be reached and the achievement of a constant flow. For each test, 60 g of dried, ground fruit was loaded into a 100 mL stainless steel extractor (SFE 20, Separex, Champigneulles, France). Each extraction run consisted of an initial static extraction phase of 30 min, followed by a dynamic extraction phase, the duration of which was determined by the specific experimental design conditions (Table 2). During the static phase, the vessel was brought to the target temperature and pressure conditions of the respective run (Table 2) without fluid flow, in order to reach dissolution equilibrium. Subsequently, the dynamic extraction was performed at a constant Sc-CO_2_ mass flow rate of approximately 1.80 g/min, corresponding to 1 L/min flow, measured under ambient temperature and pressure conditions, using a wet gas meter (Brunt, Milan, Italy). The extract from each run was collected in a Falcon tube (Falcon, Corning Life Sciences, Corning, NY, USA), recovered from the separator, and weighed, and the extraction yield was then determined.

### 3.4. Design of the Experiment and Statistical Analysis

In order to optimize the Sc-CO_2_ extraction, the three considered variables, pressure, temperature, and time, were evaluated using response surface methodology (RSM).

The RSM analysis was implemented with a central composite ratable design (CCRD), composed of three factors (*n* = 3), each with five degrees of variation (−α, −1, 0, +1, and +α). CCRD data are reported in Table 4 [39]. The design of experiment (DoE) consisted of 8 (2^n^) factorial points, 6 (2n) axial points (star points) positioned at an axial distance of α = 1.6818 (α = 2^n/4^), which enables an estimation of the curvature, and 5 replicates of the central point to estimate the experimental error [40,41].

The optimization process results in a matrix of 15 different conditions for the extractions, and the order of the experiments was randomly assigned. The other independent variables (e.g., particle size and flow rate) were kept constant. The central point was repeated five times (lines 15–19 of Table 2).

Sc-CO_2_ extraction was also compared to the conventional Soxhlet extraction with *n*-hexane, and the results are reported in Table 2.

The experimental results were then fitted with a second order polynomial function, to find the mathematical relationship between the independent variables analyzed and the yield:

(9) $$Y=\alpha_{0}+\sum_{i=1}^{3}\alpha_{i}x_{i}+\sum_{i=1}^{3}\alpha_{ii}x_{i}^{2}+\sum_{i=2}^{2}\sum_{j=1+1}^{3}\alpha_{ij}x_{i}x_{j}$$

where Y is the response, α_0_ is the constant, α_i_ the linear coefficient, α_ii_ the quadratic coefficient and α_ij_ the interaction coefficient, and x_i_ and x_j_ are the factors related to P, T, and t. The regression values were calculated using MATLAB R2025a.

### 3.5. Determination of Fatty Acid Profile via GC-FID

The fatty acid composition was determined via HRGC chromatography with an Agilent 8860 GC System chromatograph (Agilent Technologies, Santa Clara, CA, USA), having a flame ionization detector and a capillary column Excellence SGE 30QC2/BPX70. The crude extract was transesterified with methanol to obtain the corresponding fatty acid methyl esters (FAMEs). An amount of 5–20 mg of each sample was dissolved in 1 mL of *n*-heptane, and 0.5 mL of a 2N methanolic potassium hydroxide solution was added. The mixture was stirred for 2 h with a magnetic stirrer and subsequently for 1 min with a vortex mixer (VWR, Analog Vortex Mixer). After centrifugation for 5 min at 15,000 rpm, the surface layer was separated and injected directly into the gas chromatograph using an autosampler set at 250 °C in split mode (1:120–1:20 split ratio depending on the different concentrations). A gradient temperature was used: 165 °C for 5 min, increased to 240 °C at a heating rate of 5 °C/min, then maintained for 1 min and subsequently increased from 240 °C to 255 °C at a rate of 15 °C/min. The temperature for the detector was set to 260 °C. The fatty acid composition was determined using a standard reference mixture (Mix C8-C24 CRM 18918 Supelco) containing 14 fatty acid methyl esters for the identification and quantification of FAMEs in the extracts.

All chromatographic analyses of the standard reference mixture and samples were performed in triplicate (*n* = 3).

The standard mixture was injected using the same gradient temperature to define the elution time of the fatty acids that eluted, as reported in Table 12.

**Figure 10:**
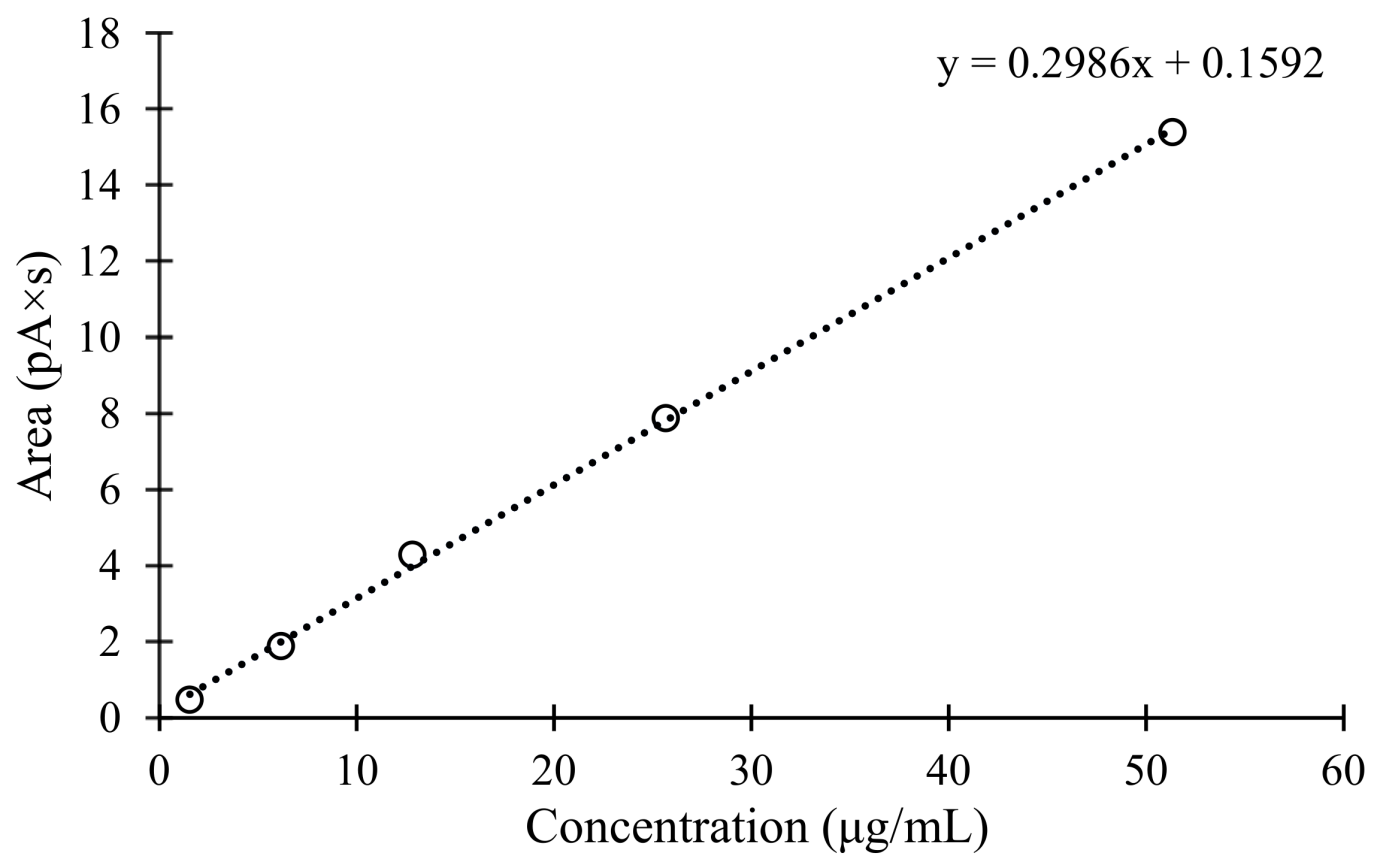
Calibration curve for methyl palmitate in the concentration range 1.5 μg/mL–50 μg/mL and corresponding linear equation.

The analytical methodology was targeted at determining the relative percentage composition (%*w*/*w*) of FAMEs. A calibration curve was established for methyl palmitate, as a representative FAME within the samples, to evaluate the instrumental response and verify system linearity. The calibration curve was constructed using pure palmitate methyl ester in a range of concentrations between 1.5 μg/mL and 50 μg/mL, which showed a good response linearity with a correlation coefficient (r^2^) of 0.999 (Figure 10).

To estimate method sensitivity, the LOD and LOQ were first determined for methyl palmitate, using the calibration curve slope, resulting in an LOD of 0.45 μg/mL and an LOQ of 1.5 μg/mL. As an indication of the estimated instrumental sensitivity over the entire analyzed mixture profile, the corresponding LOD and LOQ values for the remaining FAMEs were calculated via conversion using the respective response factors (Rf_i_, calculated as described in Equation (10)); the results are reported in Table 13. These values serve as an estimate of sensitivity rather than as multi-point calibration limits for a single compound.

For each fatty acid methyl ester in the standard mixture, a response factor (Rf_i_) was defined as indicated in Equation (10).

(10) $${Rf}_{i}=\frac{m_{i}\times \sum A_{i}}{A_{i}\times \sum m_{i}}$$

where Rf_i_ is the response factor for each fatty acid, m_i_ is the mass percentage of component i-th of the standard, and A_i_ is the area under the peak of the i-th FAME in the standard mixture. The fatty acid composition of the different extracts was determined by comparing retention times with those of standard reference mixture compounds. Complete baseline separation of all target analytes was visually confirmed through chromatograms; any adjacent peaks were well resolved without overlap or baseline interference. To establish operational integration thresholds, chromatograms obtained from blank injections were analyzed, and the mean blank signal and corresponding standard deviation (σ) were determined at the retention time of each FAME. The LOD was calculated as the mean blank signal plus three times the standard deviation (3σ) and the LOQ as the mean blank signal plus ten times the standard deviation (10σ) [42]. For each sample analyzed, individual peak areas were compared against these LOD and LOQ thresholds. For peaks exceeding the LOQ, quantification was calculated (expressed as a relative percentage composition, %_i_ *w*/*w*) following Equation (11):

(11) $${\%}_{i}=\frac{{Rf}_{i}\times A_{i}}{\sum \left({Rf}_{i}\times A_{i}\right)}\times 100$$

where %_i_ is the mass percentage of the i-th FAME in the sample, Rf_i_ is the response factor of the i-th FAME, and A_i_ is the area under the peak of the i-th FAME.

### 3.6. NMR Analysis of the Extract

^1^H and ^13^C NMR spectra were recorded with a Varian 500 spectrometer using CDCl_3_ as the solvent (residual solvent peak, δ = 7.26 ppm (^1^H NMR)/77.16 ppm (^13^C NMR) for CDCl_3_ was used as the internal standard).

^1^H NMR (500 MHz, CDCl_3_) δ 5.41–5.29 (m, 9H), 5.26 (tt, *J* = 5.9, 4.3 Hz, 1H), 4.29 (dd, *J* = 11.9, 4.3 Hz, 2H), 4.14 (dd, *J* = 11.9, 6.0 Hz, 2H), 2.80–2.73 (m, 4H), 2.41–2.25 (m, 7H), 2.10–1.96 (m, 12H), 1.62 (dtd, *J* = 13.3, 6.3, 3.8 Hz, 8H), 1.40–1.20 (m, 50H), 0.88 (td, *J* = 7.0, 4.8 Hz, 10H).

### 3.7. Antimicrobial Activity

#### 3.7.1. Microbial Strains

Four microbial strains obtained from the American Type Culture Collection (ATCC) (Manassas, VA, USA) were used in this study: *Candida albicans* (*C. albicans*) ATCC 10231, *Escherichia coli* (*E. coli*) ATCC 25922, *Pseudomonas aeruginosa* (*P. aeruginosa*) ATCC 9027, and *Staphylococcus aureus* (*S. aureus*) ATCC 6538. *C. albicans* was stored in Sabouraud Dextrose Broth (SDB, Sial group, Rome, Italy), while *E. coli*, *P. aeruginosa*, and *S. aureus* were stored in Tryptic Soy Broth (TSB, Oxoid S.p.A, Milan, Italy), both supplemented with 20% (vol./vol.) glycerin at −80 °C until use.

#### 3.7.2. Disk Diffusion Assay

The preliminary determination of the antimicrobial activity of CM oil, obtained from CM extracts 23 and 24, against all tested strains was carried out using the agar disk diffusion assay, according to the standard procedure of the Clinical and Laboratory Standards Institute [43], using pooled samples to ensure sufficient mass for testing.

Sterile discs, 6 mm in diameter, containing 10 μL of each extract resuspended in 20% dimethyl sulfoxide (DMSO), were placed on Sabouraud Dextrose Agar (SAB, Sial group, Rome, Italy) or Mueller Hinton Agar (MHA, Oxoid S.p.A, Milan, Italy) plates, previously inoculated with 100 μL of 10^7^–10^8^ CFU/mL microbial suspensions. A sterile disc supplemented with Amphotericin B and Levofloxacin served as a positive control. The potential activity of DMSO at the concentration used to resuspend the extracts (20%) was also assessed. After incubation at 37 °C for 24 h, the antagonistic activity of the extracts was quantified based on a clear zone of inhibition in the indicator meadow around the discs, and the diameters of these zones in mm were measured [44]. The disk diffusion agar test was repeated 3 times.

#### 3.7.3. The Minimum Inhibitory Concentration (MIC) and Minimum Fungicidal/Bactericidal Concentration (MFC/MBC)

The minimum inhibitory concentration (MIC) values of antimicrobials, including the positive controls Amphotericin B and Levofloxacin, were determined against all tested strains, using the broth microdilution method in 96-well microplates, according to the Clinical Laboratory Standards Institute (CLSI) guidelines 2019 [45].

The test to obtain the MIC and minimum fungicidal/bactericidal concentration (MFC/MBC) values of the *Cornus mas* L. oil extracts was performed in sterile 96-well microplates. Serial two-fold dilutions of each *Cornus mas* extract were prepared in the appropriate culture medium. Aliquots (100 µL) of each dilution were dispensed into sterile 96-well microplates. For each extract dilution, a solvent-matched control containing the same final concentration of DMSO, but without the extract, was prepared and tested in parallel. The DMSO controls were subjected to the same two-fold dilution scheme used for the extracts. Moreover, the negative control consisted of medium alone (SAB or TSB for *C. albicans* and bacteria, respectively). Then, 95 µL of Sabouraud Dextrose Broth (SDB) or Tryptic Soy Broth (TSB), depending on the microorganism tested, and 5 µL of the microbial suspension were added to each well, resulting in a final volume of 200 µL, at a concentration of 10^6^ CFU/mL. The plates were then incubated at 37 °C for 24 h. Antimicrobial activity was attributed to the extract only when microbial growth inhibition was observed relative to the corresponding solvent-matched control.

Following the MIC determination, 2,3,5-triphenyltetrazolium chloride (TTC) solution at a concentration of 0.5% (*w*/*v*) was added at a volume of 20 μL to each well to evaluate microbial metabolic activity. Wells showing no color development after 2 h of incubation at 37 °C were considered to represent the bactericidal/fungicidal endpoint under the experimental conditions employed. Since no subculture on antimicrobial-free agar was performed, this assessment was based on the absence of detectable metabolic activity. The experiment was conducted in triplicate.

#### 3.7.4. Growth Kinetics Study

The kinetics of growth of all microbial strains tested were determined by calculating the change in the optical density (OD) of microbial cultures in the presence of the *Cornus mas* L. extracts 23 and 24, used at the MIC values. In a 96-well sterile microplate, 90 μL of SDB or TSB was mixed with 100 μL of both extract 23 and extract 24 at the MIC value and with 10 μL of each microorganism with a final microbial concentration of 10^4^ CFU/mL. Measurements were performed at 1 h intervals for a total of 24 h of incubation at the optical density (OD) of 595 nm for bacteria and 540 nm for *C. albicans*, by using an automatic Tecan Sunrise™ microplate reader (Sunrise, Tecan Group Ltd., Männedorf, Switzerland). The experiment was conducted in duplicate.

#### 3.7.5. Statistical Analysis

The statistical analysis was performed using the GraphPad Prism 10.4.1. (San Diego, CA, USA) program. The statistical analysis of kinetic data was performed following the “GraphPad guide to comparing dose–response or kinetic curves” [46]. For each kinetic curve, the Area Under the Curve (AUC) was calculated to summarize the curve into a single value. Then, statistically significant differences between the experimental groups were calculated using One-way ANOVA followed by Bonferroni’s multiple comparison test. Values were considered significant at *p* < 0.05. 

## 4. Conclusions

Supercritical CO_2_ extraction applied to *Cornus mas* fruits under different operating conditions of pressure (P), temperature (T), and extraction time (t) enabled the recovery of lipophilic fractions rich in bioactive fatty acids. The obtained extracts were mainly characterized by the presence of linoleic acid (58–63%), oleic acid (22–24%), palmitic acid (8–9%), stearic acid (2–4%), arachidic acid (about 2%), and behenic acid (about 1%), as determined via HRGC analysis. The highest extraction yield (2.89%) was achieved at 270 bar, 48 °C, and 4.85 h, corresponding to almost twice the yield obtained using conventional Soxhlet extraction (1.55% in 6 h of extraction). In addition to higher extraction yields achieved in shorter extraction times, supercritical CO_2_ extraction demonstrates practical and operational advantages over the traditional Soxhlet method, including the complete elimination of toxic organic solvents and lower thermal impacts, which preserves lipophilic compounds, highlighting its strong potential for industrial scalability. These findings confirm the effectiveness of supercritical CO_2_ extraction as a green and selective technology for the recovery of lipophilic natural products from food-derived plant matrices. Furthermore, the obtained extracts demonstrated antimicrobial potential in broth microdilution assays against the tested microorganisms. In contrast, agar diffusion assays showed only limited inhibition, consistent with the restricted diffusion of lipophilic compounds in solid media. Differences observed in the antibacterial activity against *E. coli* may be related to variations in the linoleic acid content, although synergistic effects among the different fatty acids present in the extracts cannot be excluded. Although the extracts exhibited antimicrobial potential in broth microdilution assays, the present study does not allow the attribution of this activity to individual fatty acids. The observed effects are more appropriately interpreted as resulting from the overall composition of the lipophilic extract, and further studies will be required to identify the bioactive components and their mechanisms of action. Overall, these findings indicate that supercritical CO_2_ extracts of *Cornus mas* fruits represent a promising source of lipophilic bioactive compounds. Thanks to their lipid profile and proven inhibitory activity against microbial strains, the Sc-CO_2_ extracts show promising practical applications as an edible oil, a functional food ingredient, or a natural food preservative, as well as an ingredient in natural antimicrobial formulations. Future research should address current limitations by performing a more in-depth chemical characterization (e.g., LC-MS or GC-MS determination of minor lipophilic components), evaluating the oil’s long-term stability during storage, and assessing its techno-economic feasibility at a pilot scale.

## Figures and Tables

**Table 1:** Supercritical CO_2_ extraction conditions and yields for *Cornus mas* and comparable drupe seed/kernel matrices reported in the literature.

Matrix	Fruit Part	Variables Investigated	P (bar)	T (°C)	Time	Yield %	Experimental Design	Ref.
Cornelian cherry (*Cornus mas* L.)	Seeds	P, T	119–330	36–63	Fixed (90 min)	2.35–5.18	CCRD	[12]
Peach (*Prunus persica*)	Kernels	P, T, flow rate	120–160	30–40	Not reported	38.68–47.14	Box–Behnken design	[16]
Cherry (*Prunus avium*)	Seeds	P, T, flow rate, extr. time	200–350	40–70	15–240 min	Not reported	Box–Behnken design	[17]
Sour cherry (*Prunus cerasus* L.)	Seeds	P, T, flow rate	200–350	40–70	240 min	2.50–13.02	One factor at a time design	[18]
Apricot (*Prunus armeniaca)*	Kernels	P, T, flow rate, ethanol conc.	300–450	40–60	15 min	4.2–21.7	Box–Behnken design	[19]
Cornelian cherry (*Cornus mas* L.)	Whole fruits	P, T, extr. time	110–310	32–52	60–360 min	0.40–2.93	CCRD	Present work

T: temperature; P: pressure; extr. time: extraction time.

**Table 2:** Central composite design and results of experimental Sc-CO_2_ extractions.

No.	x_1_ (P)	x_2_ (T)	x_3_ (t)	P (bar)	T (°C)	t (h)	Yield%
1	−1	−1	−1	150	36	2	0.66
2	−1	−1	1	150	36	5	2.79
3	−1	1	−1	150	48	2	0.82
4	−1	1	1	150	48	5	1.70
5	1	−1	−1	270	36	2	1.59
6	1	−1	1	270	36	5	2.45
7	1	1	−1	270	48	2	2.02
8	1	1	1	270	48	5	2.93
9	−1.68	0	0	110	42	3.5	1.37
10	1.68	0	0	310	42	3.5	2.42
11	0	−1.68	0	210	32	3.5	1.02
12	0	1.68	0	210	52	3.5	2.47
13	0	0	−1.68	210	42	1	0.40
14	0	0	1.68	210	42	6	2.71
15	0	0	0	210	42	3.5	2.30
16	0	0	0	210	42	3.5	2.07
17	0	0	0	210	42	3.5	2.36
18	0	0	0	210	42	3.5	2.24
19	0	0	0	210	42	3.5	1.87
20 ^a^	-	-	-	270	48	4.85	2.96
21 ^a^	-	-	-	270	48	4.85	2.89
							
Soxhlet							1.55

^a^ Extracts 20 and 21 correspond to the independent replicates of the best extraction conditions predicted by the DoE model within the investigated range.

**Table 3:** Regression coefficients and respective *p*-values of the quadratic model (Equations (1) and (9)).

Regression Coefficients of Equation (1)	Estimate	*p*-Value
α_0_	2.1616	**2.44 × 10^−7^**
α_1_	0.35042	**0.005**
α_2_	0.1771	0.096
α_3_	0.63445	**9.38 × 10^−5^**
α_11_	−0.061396	0.536
α_22_	−0.11442	0.261
α_33_	−0.18157	0.089
α_12_	0.23	0.098
α_13_	−0.155	0.245
α_23_	−0.15	0.260

In bold if significant at a 95% confidence interval.

**Table 4:** Coded and uncoded levels of the three independent variables considered for the CCRD design.

Independent Variables		Coded Levels				
		−α	−1	0	1	α
x_1_: pressure (bar)	P	110	150	210	270	310
x_2_: temperature (°C)	T	32	36	42	48	52
x_3_: time (h)	t	1	2	3.5	5	6

**Table 5:** Regression coefficients and respective *p*-values of the reduced model (Equations (2) and (3)).

Regression Coefficients of Equation (2)	Estimate	*p*-Value
α_0_	1.9047	**1.44 × 10^−12^**
α_1_	0.35042	**0.0078**
α_3_	0.63445	**4.85 × 10^−5^**

In bold if significant at a 95% confidence interval.

**Table 6:** Quadratic and reduced model for extraction yield.

Quadratic Model	Equation	R^2^	R^2^_adj_	R^2^_pred_
Y = 2.1616 + 0.3504x_1_ + 0.1771x_2_ + 0.6345x_3_ − 0.0614x_1_^2^ − 0.1144x_2_^2^	(1)	0.8890	0.7780	−0.1968
− 0.1816x_3_^2^ + 0.2300x_1_x_2_ − 0.1550x_1_x_3_ − 0.1500x_2_x_3_				
Reduced model				
Y = 1.9047 + 0.3504x_1_ + 0.6345x_3_	(3)	0.7166	0.6755	0.6223

**Table 7:** Assignment of ^1^HNMR spectrum signals (Figure 4) for extract number 20.

NMR Peak	Associated Group	Peak Multiplicity	Peak Area	δ (ppm)
1	-CH_3_	m	9	0.90–0.85
2	-CH_2_-	m	54	1.37–1.22
3	-CH_2_-CH_2_-CO_2_-	m	6	1.64–1.57
4	-C-CH_2_-C=C-	m	10	2.06–1.98
5	-CH_2_-CO_2_-	m	6	2.34–2.29
6	-C=C-CH_2_-C=C-	t	3.763	2.78–2.74
7	-C=C-CH_2_-C=C-CH_2_-C=C-	m	0.252	2.81–2.78
8	CH(-CH_2_-O-CO-C)_2_	dd	2	4.16–4.12
9	CH(-CH_2_-O-CO-C)_2_	dd	2	4.31–4.27
10	CH(-CH_2_-O-CO-C)_2_	tt	1.000	5.28–5.24
11	C-HC=CH-C	m	9.192	5.40–5.30

For groups with different hydrogen atoms, the underlined atoms correspond to the observed peak multiplicity.

**Table 8:** Solution of Equations (4)–(7) and resulting fatty acid class distribution for extract No. 20.

Variable in Equation	Fatty Acid Saturation Class	Moles of Fatty Acid per Mole of Triglyceride	Percentage
x	mono-unsaturated	0.644	21.47%
y	di-unsaturated	1.8815	62.72%
z	tri-unsaturated	0.0630	2.10%
w	saturated	0.4115	13.72%

**Table 9:** Fatty acid profile of *Cornus mas* L. oils (expressed as %) obtained from extraction with Sc-CO_2_ and Soxhlet extraction with *n*-hexane. The average of the triplicate analyses is reported with the standard deviation in brackets.

No.	Methyl Octanoate	Methyl Decanoate	Methyl Laurate	Methyl Myristate	Methyl Palmitate	Methyl Palmitoleate	Methyl Stearate	Methyl Oleate	Methyl Linoleate	Methyl Arachidate	Methyl Linolenate	Methyl Behenate	Methyl Erucate	Methyl Lignocerate
1	0.009 (0.003)	0.005 (0.001)	0.007 (0.002)	0.089 (0.002)	**8.581 (0.050)**	0.019 (0.004)	**2.533 (0.006)**	**22.189 (0.017)**	**62.175 (0.015)**	**2.426** **(0.005)**	0.251 (0.003)	**1.362 (0.018)**	0.016 (0.004)	0.337 (0.009)
2	0.008 (0.000)	0.004 (0.000)	0.006 (0.000)	0.071 (0.001)	**8.265 (0.023)**	0.020 (0.003)	**2.270 (0.002)**	**22.434 (0.012)**	**63.168 (0.015)**	**2.408** **(0.000)**	0.196 (0.001)	**0.982 (0.007)**	0.018 (0.001)	0.151 (0.020)
3	0.028 (0.004)	0.018 (0.002)	0.015 (0.001)	0.129 (0.001)	**9.004 (0.030)**	0.021 (0.002)	**4.185 (0.006)**	**23.323 (0.081)**	**59.759 (0.051)**	**2.218** **(0.006)**	0.239 (0.000)	**0.793 (0.003)**	0.030 (0.001)	0.238 (0.032)
4	0.019 (0.009)	0.009 (0.001)	0.007 (0.001)	0.128 (0.001)	**9.028 (0.011)**	0.024 (0.002)	**2.942 (0.001)**	**22.212 (0.015)**	**60.993 (0.014)**	**2.332** **(0.001)**	0.275 (0.038)	**1.762 (0.005)**	0.018 (0.001)	0.252 (0.019)
5	0.020 (0.009)	0.007 (0.001)	0.008 (0.000)	0.083 (0.002)	**8.562 (0.044)**	0.016 (0.004)	**3.126 (0.022)**	**22.789 (0.069)**	**62.055 (0.078)**	**2.343** **(0.031)**	0.165 (0.002)	**0.604 (0.011)**	0.039 (0.012)	0.183 (0.025)
6	0.010 (0.003)	0.009 (0.000)	0.006 (0.000)	0.069 (0.001)	**8.110 (0.016)**	0.018 (0.001)	**2.799 (0.002)**	**23.022 (0.010)**	**62.152 (0.017)**	**2.366** **(0.004)**	0.201 (0.001)	**0.861 (0.007)**	0.024 (0.003)	0.354 (0.010)
7	0.008 (0.001)	0.004 (0.001)	0.004 (0.001)	0.071 (0.000)	**7.569 (0.024)**	0.017 (0.003)	**2.750 (0.004)**	**23.550 (0.018)**	**62.693 (0.001)**	**2.335** **(0.010)**	0.173 (0.001)	**0.600 (0.005)**	0.028 (0.003)	0.198 (0.024)
8	0.007 (0.003)	0.007 (0.003)	0.006 (0.002)	0.067 (0.001)	**8.018 (0.143)**	0.020 (0.002)	**2.652 (0.019)**	**23.245 (0.058)**	**62.677 (0.051)**	**2.337** **(0.004)**	0.165 (0.007)	**0.595 (0.027)**	0.027 (0.004)	0.176 (0.028)
9	0.015 (0.000)	0.007 (0.001)	0.009 (0.002)	0.110 (0.000)	**8.768 (0.007)**	0.021 (0.001)	**3.217 (0.006)**	**22.637 (0.007)**	**61.121 (0.008)**	**2.316** **(0.002)**	0.233 (0.002)	**1.154 (0.009)**	0.026 (0.001)	0.366 (0.004)
10	0.009 (0.001)	0.008 (0.001)	0.007 (0.001)	0.065 (0.001)	**8.053 (0.031)**	0.017 (0.002)	**2.563 (0.006)**	**23.016 (0.010)**	**62.697 (0.008)**	**2.352** **(0.004)**	0.193 (0.003)	**0.785 (0.012)**	0.025 (0.001)	0.207 (0.003)
11	0.007 (0.002)	0.005 (0.000)	0.006 (0.001)	0.081 (0.002)	**8.137 (0.040)**	0.020 (0.001)	**2.529 (0.007)**	**22.738 (0.018)**	**62.730 (0.035)**	**2.436** **(0.001)**	0.191 (0.001)	**0.856 (0.019)**	0.024 (0.004)	0.242 (0.004)
12	0.009 (0.002)	0.009 (0.003)	0.006 (0.001)	0.069 (0.006)	**8.333 (0.234)**	0.022 (0.005)	**2.656 (0.044)**	**22.950 (0.129)**	**62.011 (0.077)**	**2.280** **(0.012)**	0.227 (0.017)	**1.102 (0.113)**	0.027 (0.008)	0.298 (0.020)
13	0.008 (0.001)	0.007 (0.001)	0.004 (0.001)	0.054 (0.002)	**7.864 (0.003)**	0.018 (0.003)	**2.419 (0.002)**	**22.946 (0.010)**	**63.133 (0.012)**	**2.420** **(0.002)**	0.173 (0.001)	**0.709 (0.004)**	0.024 (0.001)	0.221 (0.002)
14	0.011 (0.004)	0.009 (0.002)	0.008 (0.002)	0.109 (0.001)	**9.066 (0.017)**	0.023 (0.001)	**3.326 (0.006)**	**22.250 (0.068)**	**61.293 (0.078)**	**2.372** **(0.001)**	0.224 (0.001)	**1.015 (0.005)**	0.020 (0.001)	0.273 (0.013)
15	0.027 (0.002)	0.046 (0.030)	0.013 (0.001)	0.103 (0.002)	**9.022 (0.100)**	0.024 (0.002)	**3.557 (0.010)**	**22.852 (0.024)**	**60.708 (0.040)**	**2.257** **(0.002)**	0.214 (0.006)	**0.883 (0.033)**	0.027 (0.002)	0.266 (0.011)
16	0.036 (0.061)	0.039 (0.018)	0.028 (0.006)	0.099 (0.009)	**8.446 (0.140)**	0.041 (0.009)	**2.667 (0.010)**	**22.873 (0.029)**	**62.095 (0.101)**	**2.346** **(0.009)**	0.194 (0.003)	**0.837 (0.023)**	0.038 (0.010)	0.263 (0.037)
17	0.010 (0.002)	0.005 (0.001)	0.007 (0.001)	0.079 (0.003)	**8.396 (0.138)**	0.021 (0.003)	**2.755 (0.013)**	**22.721 (0.054)**	**61.966 (0.045)**	**2.311** **(0.003)**	0.213 (0.003)	**1.140 (0.013)**	0.019 (0.001)	0.355 (0.014)
18	0.011 (0.003)	0.009 (0.002)	0.010 (0.002)	0.074 (0.002)	**8.429 (0.063)**	0.022 (0.001)	**2.799 (0.009)**	**22.907 (0.032)**	**62.445 (0.016)**	**2.304** **(0.005)**	0.167 (0.003)	**0.648 (0.028)**	0.019 (0.003)	0.154 (0.025)
19	0.025 (0.002)	0.011 (0.001)	0.010 (0.001)	0.110 (0.001)	**8.713 (0.010)**	0.021 (0.004)	**3.460 (0.006)**	**22.374 (0.017)**	**61.334 (0.023)**	**2.279** **(0.003)**	0.216 (0.005)	**1.099 (0.018)**	0.020 (0.004)	0.327 (0.005)
20 ^a^	0.016 (0.005)	0.011 (0.005)	0.007 (0.002)	0.068 (0.002)	**8.034 (0.055)**	0.020 (0.001)	**2.641 (0.006)**	**22.987 (0.022)**	**62.635 (0.018)**	**2.373** **(0.006)**	0.178 (0.002)	**0.712 (0.014)**	0.025 (0.003)	0.292 (0.012)
21 ^b^	0.014 (0.001)	0.011 (0.002)	0.010 (0.001)	0.095 (0.001)	**8.145 (0.158)**	0.020 (0.001)	**3.223 (0.015)**	**23.410 (0.050)**	**61.452 (0.089)**	**2.265** **(0.005)**	0.197 (0.005)	**0.807 (0.007)**	0.023 (0.006)	0.328 (0.023)
22 ^c^	0.031 (0.003)	0.012 (0.003)	0.011 (0.002)	0.117 (0.000)	**9.509 (0.009)**	0.026 (0.004)	**4.464 (0.001)**	**23.618 (0.200)**	**58.451 (0.215)**	**2.068** **(0.003)**	0.241 (0.001)	**0.934 (0.004)**	0.030 (0.004)	0.487 (0.016)
23 ^d^	0.028 (0.013)	0.029 (0.020)	0.014 (0.005)	0.091 (0.005)	**8.449 (0.023)**	0.032 (0.003)	**2.812 (0.003)**	**22.855 (0.006)**	**62.036 (0.044)**	**2.350** **(0.002)**	0.194 (0.001)	**0.874 (0.005)**	0.027 (0.004)	0.208 (0.008)
24 ^e^	0.026 (0.005)	0.013 (0.001)	0.012 (0.002)	0.106 (0.001)	**8.969 (0.022)**	0.018 (0.006)	**3.576 (0.003)**	**22.905 (0.024)**	**60.956 (0.008)**	**2.304** **(0.004)**	0.187 (0.002)	**0.691 (0.008)**	0.027 (0.001)	0.209 (0.030)

The main components are in bold in the table. ^a^ Extraction using the best predicted extraction condition 1°; ^b^ extraction using the best predicted extraction condition 2°; ^c^ extraction with Soxhlet; ^d^ extract 23 was obtained by mixing extracts 15–19; ^e^ extract 24 was obtained by mixing extracts 20–21.

**Table 10:** Agar disk diffusion assay of *Cornus mas* L. oil obtained with extract 23 and extract 24, Amphotericin B, Levofloxacin, and DMSO. The inhibition zones are indicated in mm ± SD.

Indicator Strains	* Cornus mas * L. Extract 23	* Cornus mas * L. Extract 24	Amphotericin B	Levofloxacin	DMSO
*C. albicans*	0	0	15 ± 1.3	/	0
*E. coli*	8 ± 0.4	0	/	30 ± 0.8	0
*P. aeruginosa*	0	0	/	25 ± 0.6	0
*S. aureus*	0	0	/	30 ± 1.1	0

**Table 11:** Minimum inhibitory concentration (MIC) and minimum fungicidal/bactericidal concentration (MFC/MBC) of *Cornus mas* L. extract 23 and with extract 24 (expressed as extract dilution), Amphotericin B, and Levofloxacin (both expressed in µg/mL).

	* Cornus mas * L. Extract 23		* Cornus mas * L. Extract 24		Amphotericin B (µg/mL)		Levofloxacin (µg/mL)	
Indicator Strains	MIC	MFC/MBC	MIC	MFC/MBC	MIC	MFC	MIC	MBC
*C. albicans*	1:3	1:2	1:3	1:2	2.5	5	/	/
*E. coli*	1:2	1:1	1:2	1:1	/	/	2.5	5
*P. aeruginosa*	1:2	1:1	1:2	1:1	/	/	2.5	5
*S. aureus*	1:2	1:1	1:2	1:1	/	/	2.5	5

**Table 12:** Order of elution of fatty acid methyl esters in the standard mixture.

Peak Number	Compound		Retention Time (min)
1	Methyl octanoate	C8:0	1.32
2	Methyl decanoate	C10:0	1.50
3	Methyl laurate	C12:0	1.86
4	Methyl myristate	C14:0	2.54
5	Methyl palmitate	C16:0	3.87
6	Methyl palmitoleate	C16:1	4.26
7	Methyl stearate	C18:0	6.27
8	Methyl oleate	C18:1	6.69
9	Methyl linoleate	C18:2	7.50
10	Methyl arachidate	C20:0	8.50
11	Methyl linolenate	C18:3	9.04
12	Methyl behenate	C22:0	11.68
13	Methyl erucate	C22:1	12.07
14	Methyl lignocerate	C24:0	14.10

**Table 13:** Estimated limit of detection (LOD) and limit of quantification (LOQ) of FAMEs.

FAME	LOD (μg/mL)	LOQ (μg/mL)
Methyl octanoate	0.012	0.039
Methyl decanoate	0.017	0.056
Methyl laurate	0.032	0.11
Methyl myristate	0.057	0.19
Methyl palmitate	0.45	1.5
Methyl palmitoleate	0.20	0.67
Methyl stearate	0.84	2.8
Methyl oleate	0.24	0.80
Methyl linoleate	0.37	1.2
Methyl arachidate	0.10	0.32
Methyl linolenate	0.085	0.28
Methyl behenate	0.061	0.20
Methyl erucate	0.11	0.37
Methyl lignocerate	0.074	0.25

## Data Availability

The original contributions presented in this study are included in the article. Further inquiries can be directed to the corresponding author(s).

## Abbreviations

The following abbreviations are used in this manuscript:

AK	Amikacin
AMP	Ampicillin
ATCC	American Type Culture Collection
AUC	Area Under the Curve
CAZ	Ceftazidime
CCRD	Central Composite Rotatable Design
CFU	Colony-Forming Units
CIP	Ciprofloxacin
CLSI	Clinical Laboratory Standards Institute
CM	*Cornus mas*
CN	Cephalexin
CPM	Cefepime
CRM	Certified Reference Material
CTX	Ceftriaxone
DoE	Design of Experiments
DMSO	Dimethyl sulfoxide
FA	Fatty Acid
FAME	Fatty Acid Methyl Ester
GC-FID	Gas Chromatography-Flame Ionization Detector
GC-MS	Gas Chromatography—Mass Spectrometry
GEN	Gentamycin
GOF	Goodness Of Fit
HRGC	High-Resolution Gas Chromatography
IPM	Imipenem
LC-MS	Liquid Chromatography—Mass Spectrometry
LOD	Limit Of Detection
LOQ	Limit Of Quantification
MBC	Minimum Bactericidal Concentration
MHA	Mueller Hinton Agar
MIC	Minimum Inhibitory Concentration
MFC	Minimum Fungicidal Concentration
NIT	Nitrofurantoin
NMR	Nuclear Magnetic Resonance
OD	Optical Density
PIP	Piperacillin
Rf	Response factor
RSD	Relative Standard Deviation
RSM	Response Surface Methodology
SAB	Sabouraud Dextrose Agar
Sc-CO_2_	Supercritical carbon dioxide
SD	Standard deviation
SDB	Sabouraud Dextrose Broth
TOB	Tobramycin
TSB	Tryptic Soy Broth
TTC	2,3,5-triphenyltetrazolium chloride
